# Identification of the molecular subgroups in Alzheimer's disease by transcriptomic data

**DOI:** 10.3389/fneur.2022.901179

**Published:** 2022-09-20

**Authors:** He Li, Meiqi Wei, Tianyuan Ye, Yiduan Liu, Dongmei Qi, Xiaorui Cheng

**Affiliations:** ^1^Innovative Institute of Chinese Medicine and Pharmacy, Shandong University of Traditional Chinese Medicine, Jinan, China; ^2^Institute of Chinese Medical Literature and Culture, Shandong University of Traditional Chinese Medicine, Jinan, China; ^3^School of Rehabilitation Medicine, Shandong University of Traditional Chinese Medicine, Jinan, China; ^4^Experimental Center, Shandong University of Traditional Chinese Medicine, Jinan, China

**Keywords:** Alzheimer's disease, heterogeneity, molecular subtype, transcriptomics, diagnosis

## Abstract

**Background:**

Alzheimer's disease (AD) is a heterogeneous pathological disease with genetic background accompanied by aging. This inconsistency is present among molecular subtypes, which has led to diagnostic ambiguity and failure in drug development. We precisely distinguished patients of AD at the transcriptome level.

**Methods:**

We collected 1,240 AD brain tissue samples collected from the GEO dataset. Consensus clustering was used to identify molecular subtypes, and the clinical characteristics were focused on. To reveal transcriptome differences among subgroups, we certificated specific upregulated genes and annotated the biological function. According to RANK METRIC SCORE in GSEA, TOP10 was defined as the hub gene. In addition, the systematic correlation between the hub gene and “A/T/N” was analyzed. Finally, we used external data sets to verify the diagnostic value of hub genes.

**Results:**

We identified three molecular subtypes of AD from 743 AD samples, among which subtypes I and III had high-risk factors, and subtype II had protective factors. All three subgroups had higher neuritis plaque density, and subgroups I and III had higher clinical dementia scores and neurofibrillary tangles than subgroup II. Our results confirmed a positive association between neurofibrillary tangles and dementia, but not neuritis plaques. Subgroup I genes clustered in viral infection, hypoxia injury, and angiogenesis. Subgroup II showed heterogeneity in synaptic pathology, and we found several essential beneficial synaptic proteins. Due to presenilin one amplification, Subgroup III was a risk subgroup suspected of familial AD, involving abnormal neurogenic signals, glial cell differentiation, and proliferation. Among the three subgroups, the highest combined diagnostic value of the hub genes were 0.95, 0.92, and 0.83, respectively, indicating that the hub genes had sound typing and diagnostic ability.

**Conclusion:**

The transcriptome classification of AD cases played out the pathological heterogeneity of different subgroups. It throws daylight on the personalized diagnosis and treatment of AD.

## Introduction

Alzheimer's disease (AD) is the most common type of dementia in the elderly. In 2019, the United States officially announced that AD had become the sixth leading cause of death in the United States. According to statistics, the average service cost paid by medical insurance for patients with AD and dementia is more than three times that of other older adults. The fee paid by Medicaid is 23 times (1). AD has become a significant challenge to public health.

It is always a great challenge to identify the risk population and diagnose the pathological course of AD in the preclinical stage. The Nincds-Adrad core symptom scale, introduced in 1984, has been widely used in studies with an accuracy of 65–96% but can only distinguish AD from other dementias with a specificity of 23–88% (2). In 2011, the National Institute on Aging and the Alzheimer's Association developed a new research framework combining biomarkers as an auxiliary diagnostic basis for the pathological process of AD (3). Amyloid deposition, tau protein disease, and neurodegeneration are described as an “A/T/N” system. AD is defined by its underlying pathologic processes that can be confirmed by postmortem examination of amyloid and tau pathologies. A patient that has a clinical phenotype of dementia, but does not have plaques and tangles, is categorized into other suspected AD-related dementias or non-Alzheimer. The identification of additional biomarkers may help categorize participants ante-mortem into AD vs. other dementias. The associated biological markers can be dynamically monitored by cerebrospinal fluid and special imaging (4). Recently, reliable blood detections of plaque and tangle pathology have been further developed, suggesting that blood markers may be of value in the preclinical screening of AD (5). Another possible fact is that the measures of neuron damage commonly used in AD studies, such as magnetic resonance imaging (MRI), fluorodeoxyglucose (FDG) PET, and total cerebrospinal fluid tau (T-tau), are general indicators of damage that can be caused by a variety of causes such as cerebrovascular injury (6). The 2019 Canadian Consensus Conference (CCCDTD) recommended adding other pathological factors such as vessel, inflammation, synuclein, and TDP-43 to the biological definition and questioned the significance of brain amyloid and/or tau protein (7). Between 10 and 30% of individuals clinically diagnosed with AD showed no AD-like neuropathological changes at autopsy and had normal PET-CT findings or CSF Aβ42 levels (3). The limitation of biomarkers in AD studies is that they can only indicate a subset of patients. Existing diagnostic techniques cannot achieve accurate individualized medicine at the genetic level, and molecular subtypes can provide a bridge for individually marked molecular target drugs.

Since the discovery of AD, only Selegiline, Galantamine, Rivastigmine, Memantine, Donepezil, and Aduhelm were approved by the Food and Drug Administration (FDA). However, current treatments for AD are only intended to relieve symptoms, without a cure. More early treatment may result in a more significant benefit for patients. Early diagnosis is the basis for early treatment and could contribute to AD treatment. Early diagnosis may be more benefiter to AD patients. In addition, the performance of each AD stage is affected by gene mutations and epigenetic changes, and the response to drugs also shows biological heterogeneity. The etiology and clinical heterogeneity of AD complicates the diagnosis and treatment of AD and the design and testing of new drugs (8). However, current genetic research has not yet provided preventive treatment strategies or clinical guidance for carriers of specific AD-related genetic variants (9). At present, the classification criteria based on the severity of neurological symptoms or pathological markers cannot well indicate the genetic differences among AD patients. Tumor samples distinguish subtypes by gene expression patterns, revealing the heterogeneity between tumors, guiding treatment, and predicting clinical endpoints (10). Molecular subtypes are also crucial in revealing the heterogeneity of AD. However, current studies have focused on differential gene expression between AD and non-AD (11). There are few studies on differential expression among AD patients.

In recent years, the development of high-throughput genome sequencing has enabled us to quickly analyze the genomic polymorphism of thousands of subjects, which will help us better understand AD. Yan et al. (12) systematically identified 16 co-expressed gene modules associated with AD development using WGCNA and identified six hub genes as possible biomarkers. The largest GWAS in Europe identified 20 susceptibility sites for AD (13). In the context of transcriptomics, gene network analysis can identify concurrent (or co-expressed) genes and significantly differentially expressed genes. The object of this study was to find more precise biomarkers to facilitate the early diagnosis of the disease based on transcriptomics. In this study, according to the characteristics of the gene expression profile, we divided AD cases into three subgroups. These subgroups showed different functional and clinical features. Finally, we identified the core genes of each subset, which have good diagnostic value and may provide a new strategy for treating AD.

## Methods

**Figure 1:**
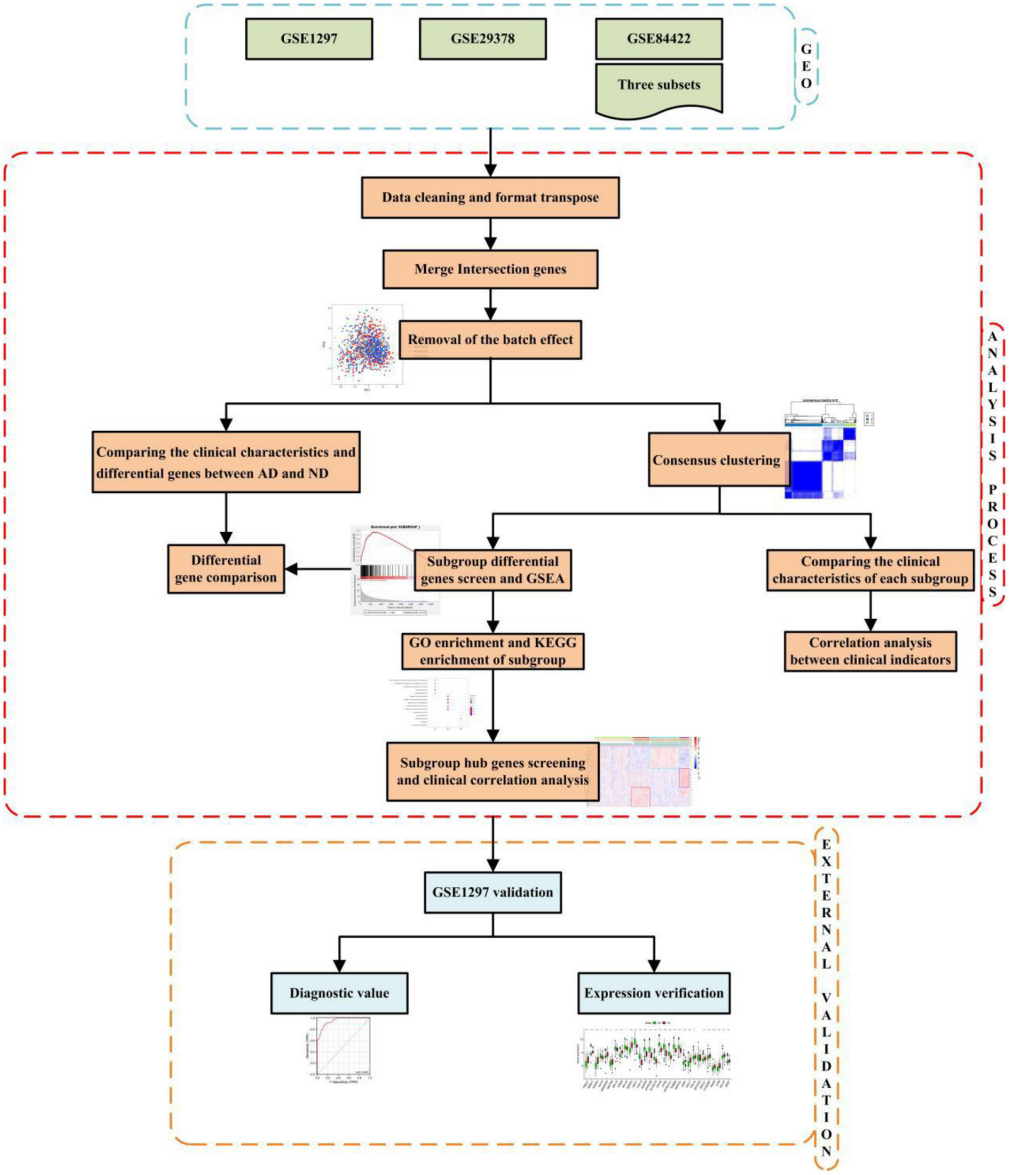
Technical strategy.

The technical strategy of this research is shown in Figure 1.

### Date collection

Three AD datasets of GSE1297 (14), GSE29378 (15), and GSE84422 (16) were acquired from the Gene Expression Omnibus (GEO) database (https://www.ncbi.nlm.nih.gov/geo/), GSE84422 includes three subsets which named GSE8442201, GSE8442202, and GSE8442203, respectively. We only apply “NO-AD (ND)” and “identified AD” in GSE84422. After data cleaning and format transposing, the files consisted of the gene expression matrix and clinical characteristics.

### Removal of the batch effect

The Intersection genes from five datasets (GSE84422 includes three subsets) were obtained and merged. We chose ComBat to normalize the expression values (17), which were log2-transformed before the cross-platform normalization.

Details of ComBat normalize:

Step 1: Standardize the data


$$\begin{array}{l} Z_{ijg}=\frac{Y_{ijg}-{\hat{\alpha }}_{g}-X{\hat{\beta }}_{g}}{{\hat{\sigma }}_{g}} \end{array}$$

Step 2: Estimated batch effect parameters

Step 2.1:Assume the parametric forms for prior distributions on the batch effect


$$\begin{array}{l} \gamma_{ig}\sim N\left(Y_{i},\tau_{i}^{2}\right)\;and\;\delta_{ig}^{2}\sim Inverse\;Gamma\;\left(\lambda_{i},\theta_{i}\;\right) \end{array}$$

Step 2.2:Calculate batch effect estimates


$$\begin{array}{l} \gamma_{ig}^{{}^{*}}=\frac{n_{i}{\bar{\tau }}_{i}^{2}{\hat{\gamma }}_{ig}+\delta_{ig}^{2^{*}}{\bar{\gamma }}_{i}}{n_{i}{\bar{\tau }}_{i}^{2}+\delta_{ig}^{2^{*}}}\;and\;\delta_{ig}^{2^{*}}=\frac{{\bar{\theta }}_{i}+\frac{1}{2}\sum_{j}{\left(Z_{ijg}-\gamma_{ig}^{{}^{*}}\right)}^{2}}{\frac{n_{j}}{2}+{\bar{\lambda }}_{i}-\;1} \end{array}$$

Step 3: Adjust the data for batch effects


$$\begin{array}{l} \gamma_{ijg}^{{}^{*}}=\frac{{\hat{\sigma }}_{g}}{{\hat{\delta }}_{ig}^{{}^{*}}}\left(Z_{ijg}-{\hat{\gamma }}_{ig}^{{}^{*}}\right)+{\hat{\alpha }}_{g}+X{\hat{\beta }}_{g}. \end{array}$$

The principal component analysis was introduced to check out whether the batch effect was removed.

### Comparing the clinical characteristics and differential genes between AD and ND

Based on the datasets to the definition of disease states, the samples were distinguished as AD or ND. The differences in Clinical dementia score (CDR), Braak, neurofibrillary tangles (NFT), CERAD, Neuritic Plaque Density (NPD), and pH between AD and ND were used pairwise Wilcoxon's rank-sum test. Benjamini-Hochberg adjusted *P* < 0.05 and the absolute difference of means >0.2 as thresholds, Wilcoxon's-sum rank test was used to test the differential expression. It is noted that the difference in means was calculated by subtracting the mean of expression in ND samples from that in samples of the AD samples.

### Consensus clustering

We use consensus clustering (18) to classify the AD samples into different subgroups. The maximum cluster number was set to 10, and the cluster consensus score >0.8 was determined to filter adjustment. All operations are performed in R4.03.

### Comparing the clinical characteristics of each subgroup

The differences in CDR, Braak, NFT, CERAD, NPD, pH, and age between subgroups and ND groups were used pairwise to Wilcoxon's rank-sum test. Furthermore, the pairwise proportion test was used to compare the proportion of women in four groups. In addition, the correlation between CDR, CERAD, Braak, NFT, NPD, Age, Gender, and pH was focused on by “Spearman” correlation analysis.

### Subgroup differential genes and gene set enrichment analysis

Benjamini–Hochberg adjusted *P* < 0.05 and the absolute difference of means >0.2 as thresholds, Wilcoxon's-sum rank test was used to test the differential expression. It is noted that the difference in means was calculated by subtracting the mean of expression in ND samples from that in samples of the subgroup. By comparing gene expression in each subgroup with other subgroups, subgroup-specific upregulated genes were identified. Moreover, the gene expression in subgroups was also compared with ND cases. We use GSEA4.1.0 software to implement gene set enrichment analysis (GSEA). Subgroup-specific genes as subgroup-specific databases. *P*-values for Student's *t*-test were calculated by comparing each subgroup with ND samples. Furthermore, the gene list for each subgroup was ranked by *P*-values.

### GO enrichment and KEGG enrichment of subgroup

GO analysis and KEGG analysis were performed for each module. In R software, the function “enrich GO” was used for GO enrichment analysis, and the database was org.Hs.eg.db (doi: 10.18129/B9.bioc.org.Hs.eg.db.). Moreover, the “enrich KEGG” function was applied for KEGG enrichment analysis (https://www.kegg.jp/). As for the parameters of the two functions, species were set to “has,” and the *q*-value was set to 0.05. The gene expression heatmap in the pathways we were interested in was also analyzed.

### Subgroup hub gene screening and clinical correlation analysis

Based on RANK METRIC SCORE in GSEA, the Top10 gene in each subgroup was screened for subsequent analysis. Vioplot, which uses the Kruskal–Wallis test described the expression of hub genes in different groups. We noted hub genes function using uniport (https://www.uniprot.org/), GeneCards (https://www.genecards.org/), and published literature. Heat maps of gene-gene correlations for the same subgroup were created by “Spearman.” The module eigengenes (MEs) based WGCNA algorithm was used to evaluate the correlation of gene subgroups with clinical traits, and the subgroups' gene expression-trait heatmaps were mapped. Furthermore, a correlation analysis between gene expression and clinical characteristics was conducted by “Spearman.”

### Diagnostic value evaluation

GSE5281 (19–22) was used to verify the diagnostic value. Boxplot, which uses the Wilcoxon's rank-sum test to verify the expression of hub genes in an external dataset. Receiver operating characteristic (ROC) curve analysis was used to investigate the value of hub genes and genes union in diagnostic efficiency to differentiate among AD and ND patients. The area under the curve (AUC) was quantified with packages (“pROC”) and packages (“glmnet”). When an AUC value was higher than 0.9, the hub genes were regarded as having outstanding specificity and sensitivity. When an AUC value was in the range of 0.7–0.9, the hub genes were regarded as having specificity and sensitivity.

## Results

### Characteristics of datasets

Clinical features and gene expression data were fetched from the GEO database, and a total of 1,240 brain samples, including 743 AD samples and 497 ND samples from three independent studies were analyzed. It covered GSE1297 (14), GSE29378 (15), and GSE84422 (16), which were processed by Affymetrix Human Genome U133A Array, Affymetrix Human Genome U133B Array, Illumina HumanHT-12 V3.0 expression bead chip, and Affymetrix Human Genome U133 Plus 2.0 Array. CDR, Braak, NFT, CERAD, NDP, pH, age, and gender were provided in terms of clinical features.

### Removal of different datasets batch effect

**Figure 2:**
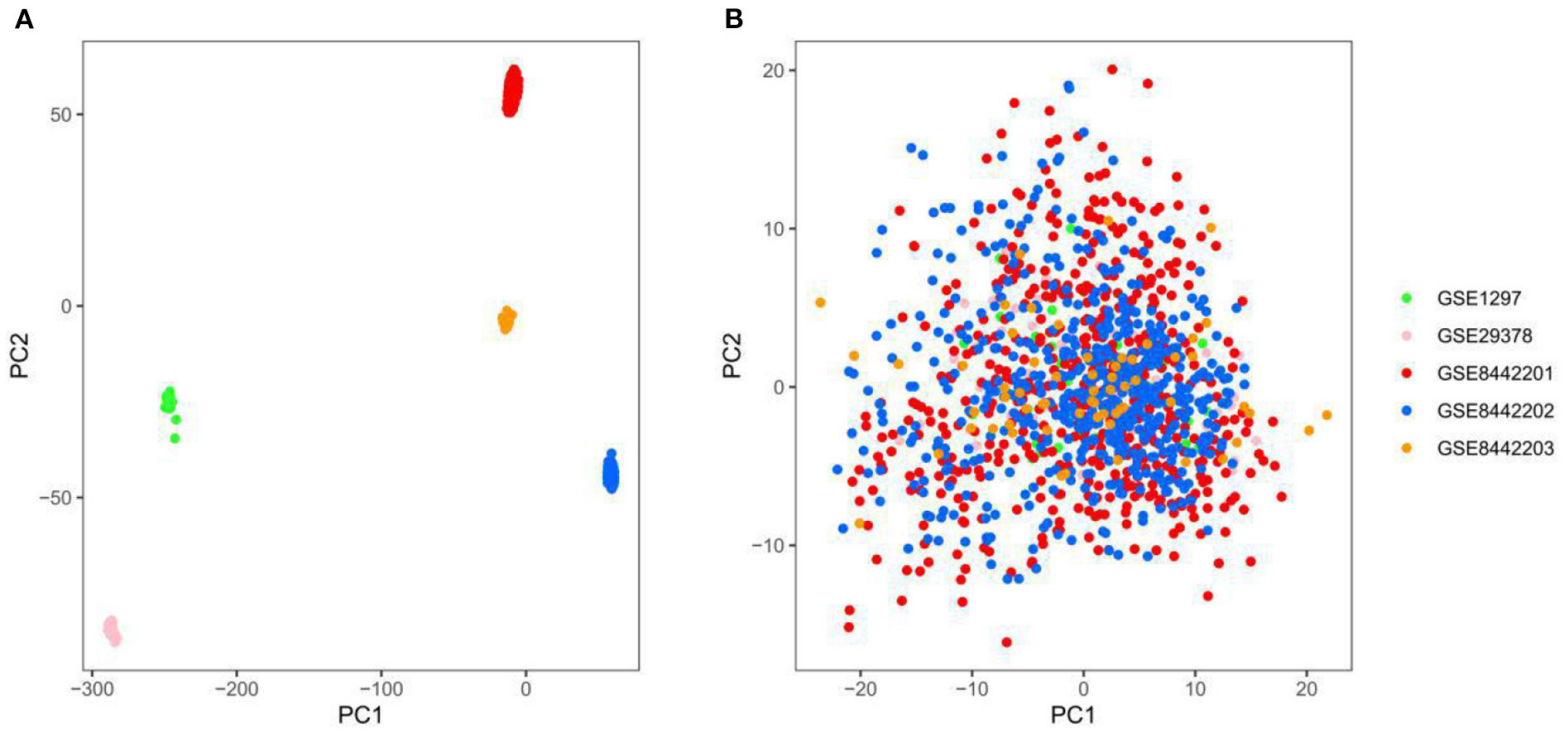
Principal component analysis (PCA) of the gene expression datasets, different colors represent specific samples. **(A)** Samples were visualized before removing the batch effect. **(B)** Samples were visualized after removing the batch effect. GSE1297, *n* = 31; GSE29378, *n* = 63; GSE8442201, *n* = 542; GSE8442202, *n* = 542; GSE8442203, *n* = 62.

Three thousand seven hundred forty-five genes were detected after the intersection of genes from different datasets was obtained and merged. The ComBat method was used to remove batch effects between datasets, which root in different platforms and batches. We adopted principal component analysis (PCA) to verify the effect of removing batch effects. The five datasets (GSE84422 includes three subsets) were separated, which shows significant differences between them (Figure 2A). In contrast, the scatter plot based on PCA shows a random distribution of samples, which indicates that the batch effects were successfully removed (Figure 2B).

### Analysis of clinical characteristics and genetic differences between AD and ND

**Figure 3:**
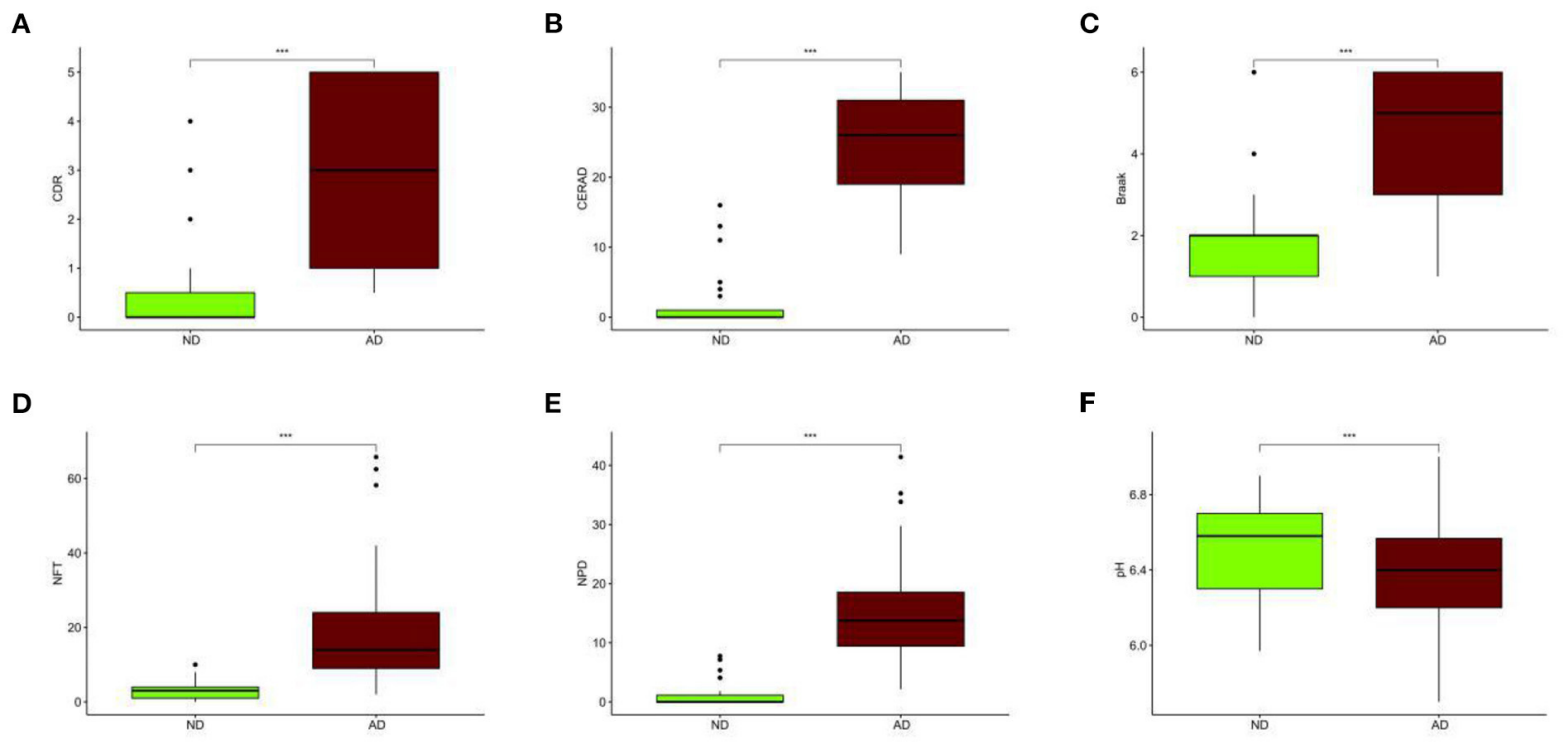
Different clinical characteristics between AD and ND. **(A)** Different CDR between AD and ND. **(B)** Different CERAD between AD and ND. **(C)** Different Braak between AD and ND. **(D)** Different NFT between AD and ND. **(E)** Different NPD between AD and ND. **(F)** Different pH between AD and ND. ND, *n* = 456–489; AD, *n* = 690–739. vs. ND, Wilcoxon's rank-sum test. ***P < 0.001.

Compared with the ND group, the CDR, CERAD, Braak, NFT, and NPD were significantly increased in the AD group (*P* < 0.001; Figures 3A–E). Furthermore, the pH of the AD group was significantly decreased (*P* < 0.001, Figure 3F). The clinical characteristics of each sample are shown in Supplementary File 1. With Benjamini–Hochberg adjusted *P* < 0.05 and the absolute difference of means >0.2 as the filtering condition, we performed differential expression analysis by comparing gene expression profiles between two groups, and 26 different genes were found (Supplementary File 2).

### Subtypes of AD samples

**Figure 4:**
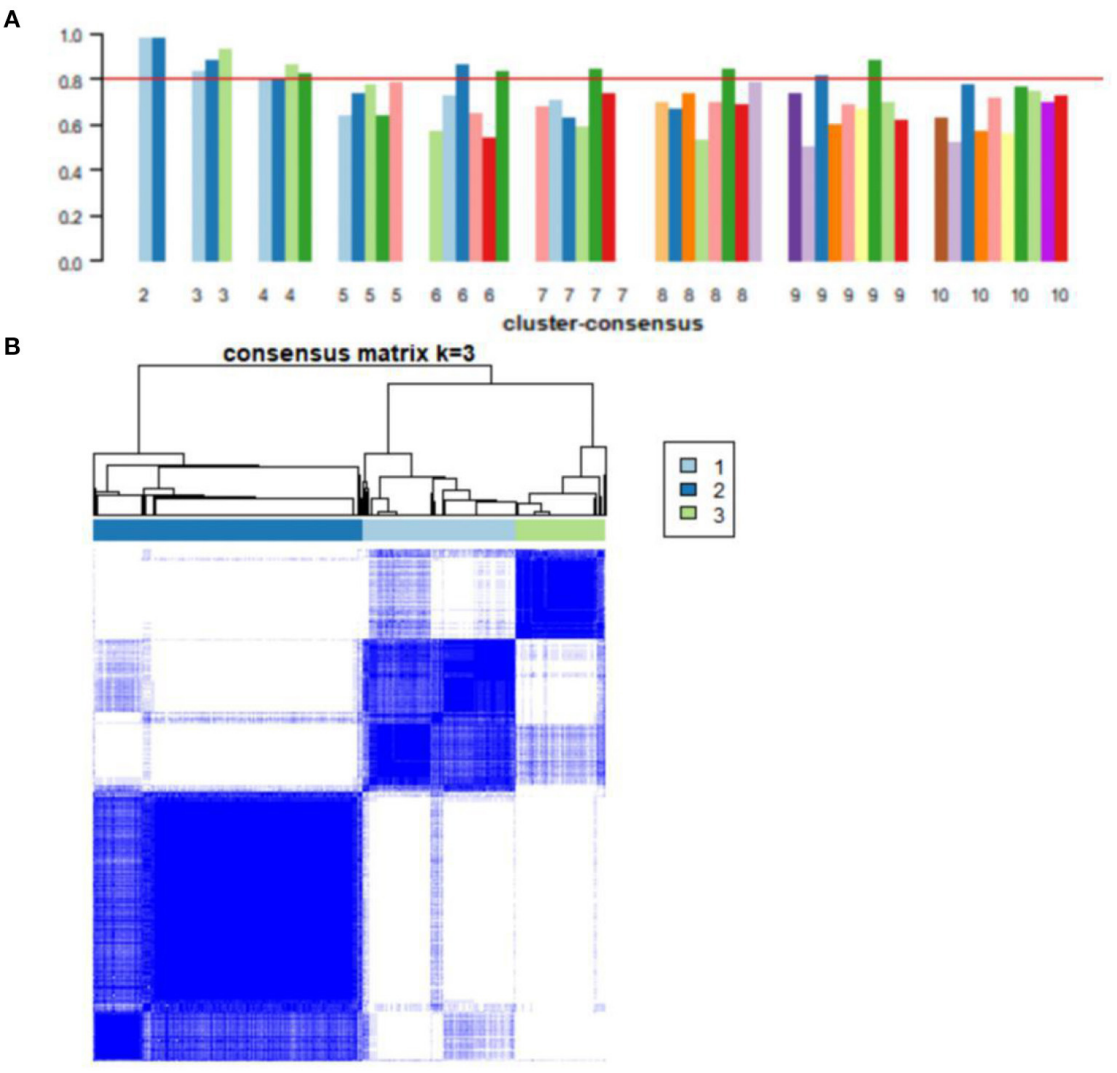
Consensus clustering analysis of gene expression profiles for AD samples. **(A)** The bar plots represent the consensus scores for subgroups with cluster counts ranging from 2 to 10. Cluster count was most stable in three subgroups, and consensus scores for subgroups >0.8. **(B)** The heat map represents the consensus matrix with a cluster count of 3.

Consensus clustering, an unsupervised method, was used to classify 743 AD samples' gene expression profiles after removing batch effect into subgroups. We divided them into two to nine subgroups (Supplementary Figure 1). The cluster consensus score suggested that compared with other subgroup classifications, the three-subgroup classification was robust. Each subgroup score was higher than 0.8 (Figure 4A). While the two-subgroup scores were also higher than 0.8, on a stable basis, more subgroups can make the analysis more detailed. Therefore, the classification of three-subgroups was selected for subsequent analysis. Moreover, based on the consensus matrix, three subgroups with 223, 391, and 129 samples in subgroups I, II, and III had highly similar gene expression patterns within each subgroup and significantly different expression patterns between each subgroup (Figure 4B). The sample clustering was shown in Supplementary File 3. Furthermore, the distribution of samples from the different GEO datasets into the three subgroups was shown in Supplementary Figure 2.

### Clinical characteristics of AD subgroups

**Figure 5:**
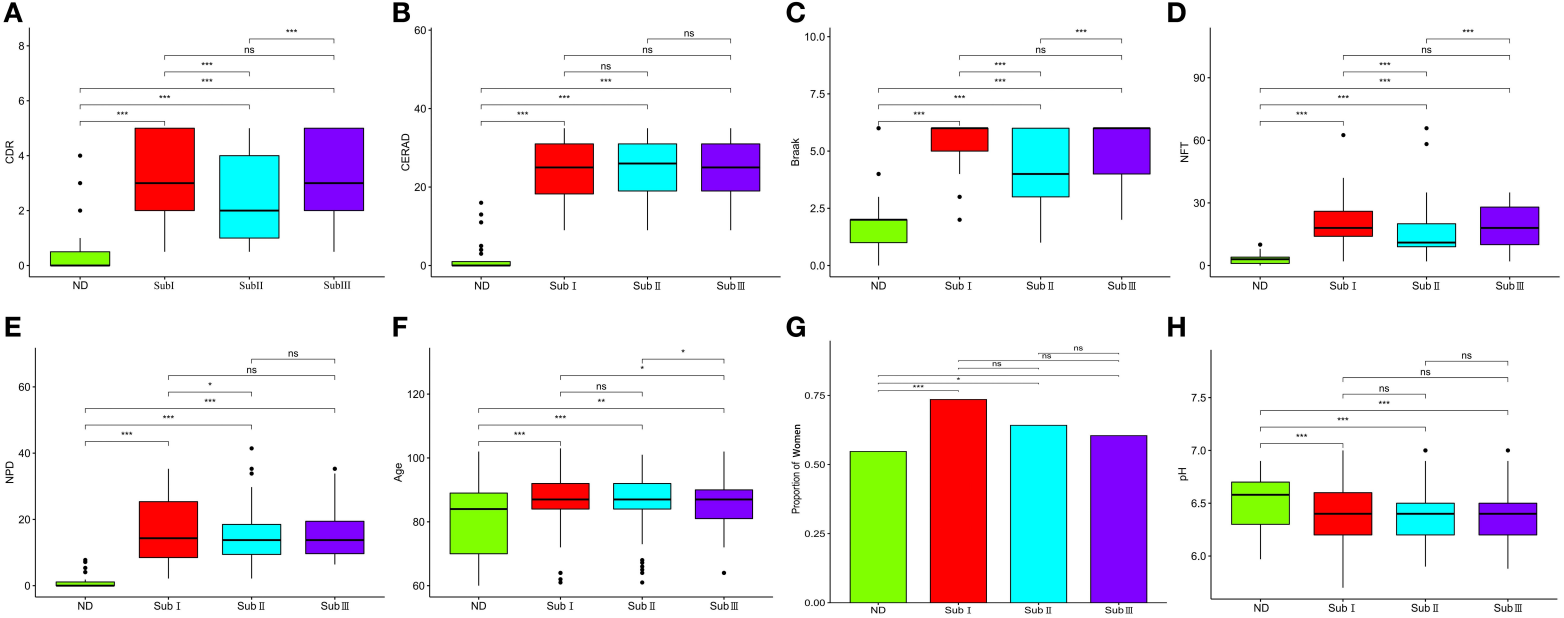
Clinical characteristics of subgroups. **(A)** Comparison of CDR between each group. **(B)** Comparison of Braak between each group. **(C)** Comparison of NFT between each group. **(D)** Comparison of CERAD between each group. **(E)** Comparison of NPD between each group. **(F)** Comparison of pH between each group. **(G)** Proportion of women. **(H)** The proportion of women in each subgroup and ND group. ND, *n* = 456–497; Sub I, *n* = 202–223; Sub II, *n* = 368–391; Sub III, *n* = 120–129. Wilcoxon's rank-sum test, ^ns^*P* > 0.05; *P < 0.05; **P < 0.01; ***P < 0.001.

**Figure 6:**
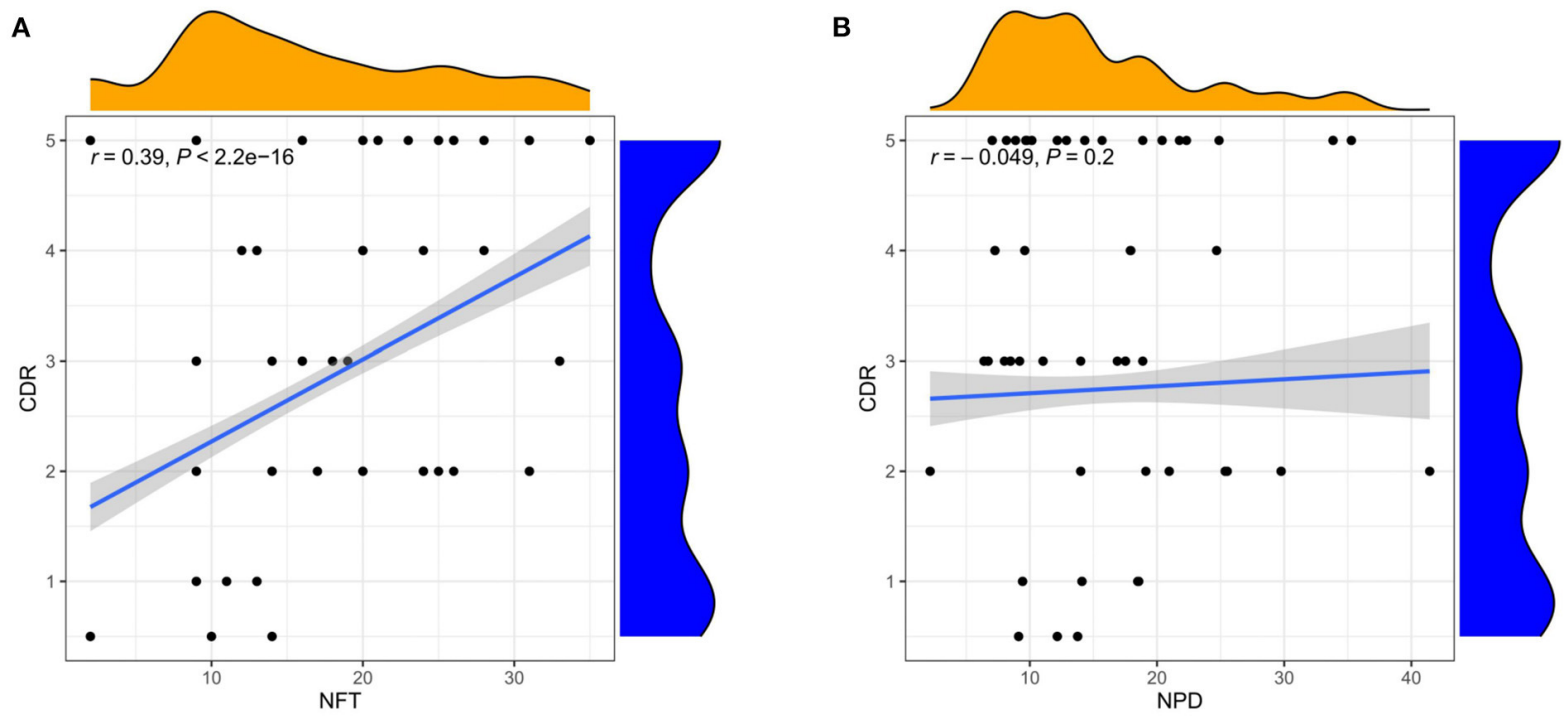
Correlation analysis of clinical features. **(A)** Correlation between CDR and NFT. **(B)** Correlation between CDR and NPD. Spearman correlation coefficient. *n* = 1,146.

The three subgroups' CDR, CERAD, Braak, NFT, NPD, and age were significantly increased in the AD group (*P* < 0.001 or *P* < 0.01; Figures 5A–F). Furthermore, the proportion of women in Subgroup I and Subgroup II were significantly higher than in ND group (*P* < 0.001 or *P* < 0.05); subgroup III had no difference from the ND group (Figure 5G). The pH of the three subgroups was lower than the ND group (*P* < 0.001, Figure 5H). Compared with subgroup II, the CDR, Braak, and NFT were high in subgroup I and subgroup III (*P* < 0.001; Figures 5A,C,D), and the NPD was increased in subgroup I (*P* < 0.05; Figure 5E). As for the CERAD, the proportion of women, and pH, there was no difference between the three subgroups (Figures 5B,G,H). The people in subgroup I and subgroup II were older than subgroup III (*P* < 0.05; Figure 5F). In addition, the correlation between CDR with NFT and NPD was focused on. We found that CDR was positively correlated with NFT while CDR was not correlated with NPD (Figures 6A,B). Supplementary Figure 3 showed the collection between CDR with CERAD, Braak, Age, Gender, and pH. The correlations of CDR with Braak and NTF persist, and no correlation between CDR and NPD in the individual subgroups. The relevant results can be obtained in Supplementary Figure 4. Furthermore, the clinical characteristics of each sample are shown in Supplementary File 1.

### Gene set enrichment analysis

**Figure 7:**
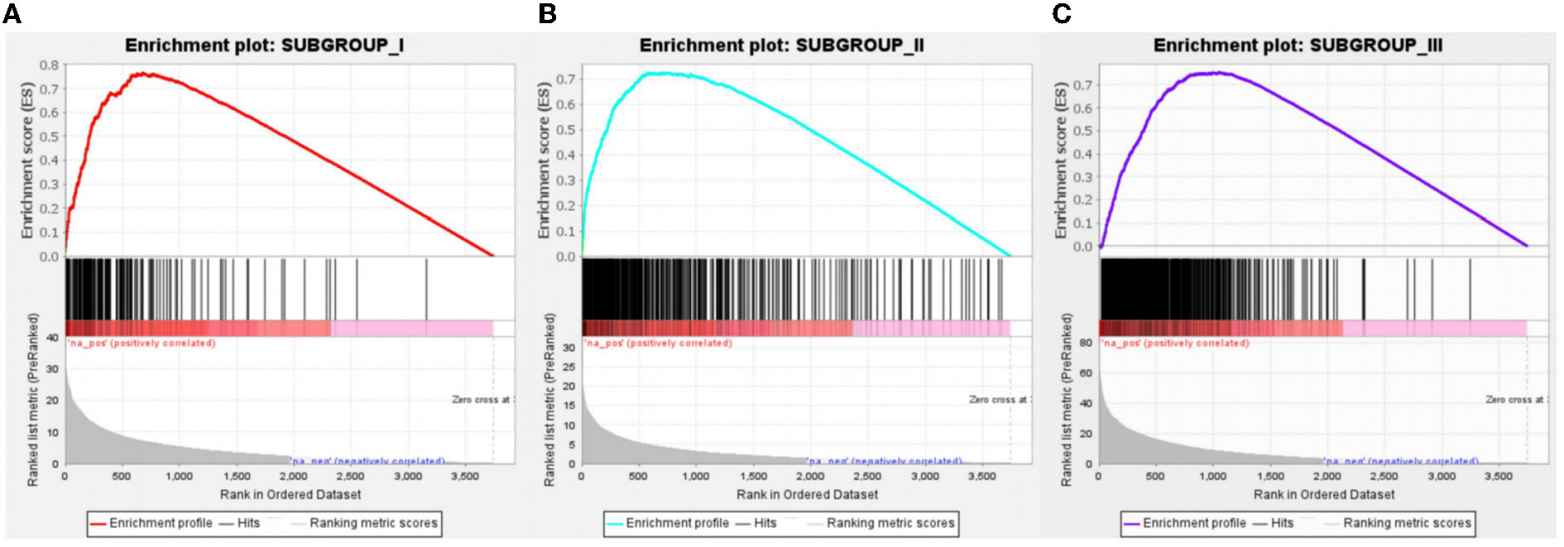
Gene Set Enrichment Analysis of subgroup-specific upregulated genes. **(A)** Compared to the ND group, specific genes in subgroup I was significantly upregulated, *n* = 149. **(B)** Compared to the ND group, specific genes in subgroup II were significantly upregulated, *n* = 403. **(C)** Compared to the ND group, specific genes in subgroup III were significantly upregulated, *n* = 491. FDR < 0.001.

With Benjamini–Hochberg adjusted *P* < 0.05 and the absolute difference of means >0.2 as the filtering condition, we performed differential expression analysis by comparing gene expression profiles between every two subgroups. One thousand forty-three subgroup-specific upregulated genes were found, there were 149,403 and 491 subgroup-specific upregulated genes in subgroup I, subgroup II, and subgroup III, respectively (Supplementary File 2). Furthermore, each subgroup with normals was also compared. There are 403, 144, and 945 different genes in subgroups, respectively (Supplementary File 2). But compared with result 3.3, in which there were only 26 differentially expressed genes between the AD and ND groups, after distinguishing subgroups, there were more different genes that appeared. This indicated that several essential genes might be ignored in previous studies that merely compare normal and disease. GSEA revealed that each subgroup specificity upregulated genes differed significantly in the ND group comparisons (FDR <0.001, Figures 7A–C). This indicates that the subgroup-specific upregulated genes can distinguish the subgroup well and distinguish the subgroup from the ND group.

### GO and KEGG enrichment analysis

**Figure 8:**
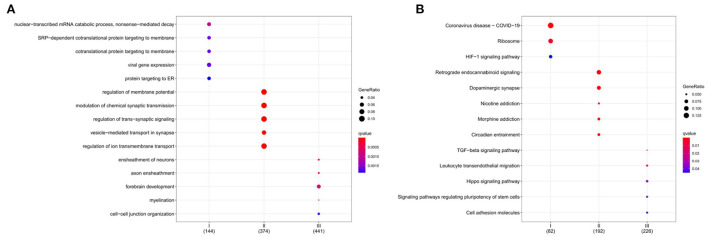
Functional characterization of a subgroup. **(A)** BP enrichment for each subgroup. **(B)** KEGG enrichment for each subgroup. The color indicates significance. The size of the circle means Gene Ratio.

GO enrichment analysis illustrates gene function on biological process (BP) levels. BP in subgroup I mainly involves nuclear-transcribed mRNA catabolic process, nonsense-mediated decay, SRP-dependent cotranslational protein targeting to membrane, and cotranslational protein targeting to the membrane. BP in subgroup II was mainly related to the regulation of membrane potential, modulation of chemical synaptic transmission, and regulation of trans-synaptic signaling. BP in subgroup III was primarily concerned with the ensheathment of neurons, axon ensheathment, and forebrain development (Figure 8A). According to KEGG enrichment results, subgroup I mainly involved Coronavirus disease—COVID-19, Ribosome, and HIF-1 signaling pathway; subgroup II was mainly related to Retrograde endocannabinoid signaling, Dopaminergic synapse, and Nicotine addiction; subgroup III was primarily concerned with TGF-beta signaling pathway, Leukocyte transendothelial migration, and Hippo signaling pathway (Figure 8B). Furthermore, the gene expression in the pathways also was attention. All details can be found in Supplementary Files 4, 5.

### The clinical correlation analysis of hub genes in subgroups

**Figure 9:**
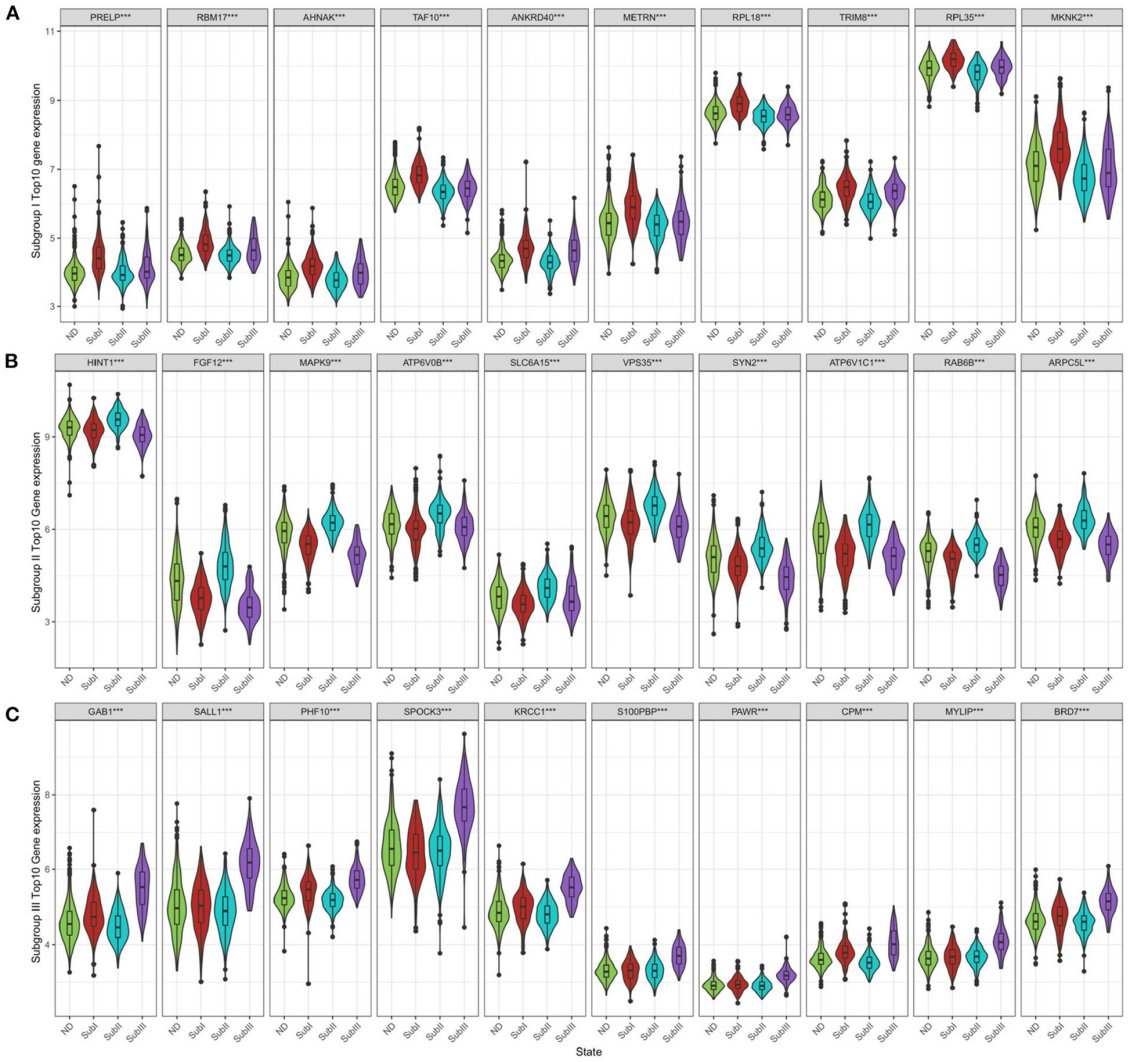
Subgroup-specific gene expression. **(A)** Expression of subgroup I hub genes in each group. **(B)** Expression of subgroup II hub genes in each group. **(C)** Expression of subgroup III hub genes in each group. Kruskal–Wallis test, ***P < 0.001.

**Figure 10:**
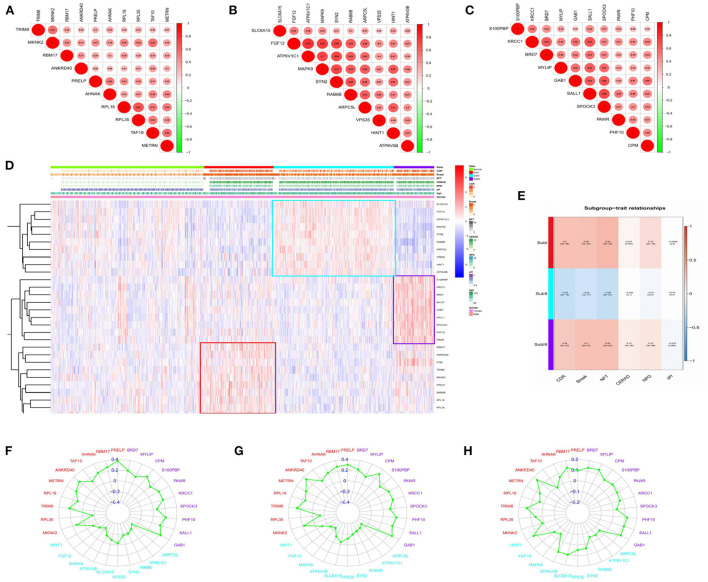
Correlation analysis. **(A)** Gene-to-gene interactions in subgroup I. **(B)** Gene-to-gene interactions in subgroup II. **(C)** Gene-to-gene interactions in subgroup III. The size and color of the circle reflect the magnitude of the interaction, with larger size and darker color illustrating that the interaction has been reinforced. **(D)** Clinical relevance heatmap of single-gene. Red represents high expression. Blue represents low expression. White means no difference. **(E)** Subgroup-trait relationship of combination-gene in each subgroup. Red represents high expression. The number above the parentheses represents the correlation. The number in parentheses represents the *P*-values. Blue represents low expression. White means no difference. **(F)** Correlation analysis between hub genes and CDR in each subgroup. **(G)** Correlation analysis between hub genes and NFT in each subgroup. **(H)** Correlation analysis between hub genes and NPD in each subgroup.

We selected the top 10 genes of groups for follow-up studies, according to CORE ENRICHMENT of GSEA (Supplementary File 6). Figure 9 showed the expression of hub genes in ND and AD subgroups. We noted gene function through uniport (https://www.uniprot.org/), GeneCards (https://www.genecards.org/), and published literature (Table 1). Gene correlation heatmaps show gene-to-gene interactions in each subgroup (Figures 10A–C). In order to study the correlation between TOP10 genes and clinical characteristics, we mapped the clinical relevance heatmap of single-gene and combination-gene in each subgroup (Figures 10D,E). Existing clinical indicators such as CDR, Braak, NFT, CERAD, and NPD can only distinguish between AD and ND. They cannot directly define the AD caused by the status change of different functions. In comparison, our molecular subtypes could define the AD state caused by different functional impairments (Figure 10D). Subgroup I and subgroup III were positively correlated with major clinical indicators such as CDR, Braak, NFT, and NPD. In contrast, subgroup II was negative (Figure 10E). Furthermore, the correlation between hub genes with CDR, NFT, and NPD was focused on (Table 1, Figures 10F–H).

**Table 1:** Functional annotation of hub genes.

**Subgroup**	**Gene name**	**Protein**	**Correlation with CDR**		**Correlation with NFT**		**Correlation with NPD**		**Function**
			***r***	***P*** **-value**	***r***	***P*** **-value**	***r***	***P*** **-value**	
I	PRELP	Prolargin	0.37	2.20E-16	0.3	2.20E-16	0.096	1.10E-03	Prolargin is a member of the small leucine-rich proteoglycan (SLRP) family, binding the basement membrane heparan sulfate proteoglycan perlecan, and triple-helical collagens type I and type II was identified in rat pituitary folliculostellate cells and capillary pericytes in expression (23). Play a role in spinal degenerative diseases (24).
	RBM17	Splicing factor 45	0.27	2.20E-16	0.28	2.20E-16	0.13	1.50E-05	Involved in the regulation of alternative splicing and the utilization of cryptic splice sites. RBM17 dismiss can lead to rapid degeneration of the Purkinje nerve and early embryonic death, which may be related to neuron survival (25). Contributing to cancers, viral infections, and the development of neurological disorders (26).
	AHNAK	Neuroblast differentiation-associated protein AHNAK	0.22	1.10E-13	0.22	5.90E-14	0.068	0.021	May be required for neuronal cell differentiation. Participate in the regulation of RNA splicing, calcium steady-state, voltage gating calcium channel activity. AHNAK is a differentiated protein for normal aging and AD brains and may act as a new biomarker for age-related neurodegenerative changes (27). At the same time, it is involved in a series of physiological activities such as immune regulation, cell structure maintenance, and neuronal excitability (28).
	TAF10	Transcription initiation factor TFIID subunit 10	0.13	1.50E-05	0.14	1.20E-06	−0.0089	0.76	TIIFD is a multimeric protein complex that plays a central role in mediating promoter responses to various activators and repressors.
	ANKRD40	Ankyrin repeat domain-containing protein 40	0.27	2.20E-16	0.34	2.20E-16	0.16	3.20E-08	It may be associated with neurodevelopmental disorders (29).
	METRN	Meteorin	0.21	6.60E-13	0.21	3.30E-13	0.067	0.023	Involved in both glial cell differentiation and axonal network formation during neurogenesis. A neurotrophic factor that regulates angiogenesis (30, 31).
	RPL18	60S ribosomal protein L18	0.11	2.30E-04	0.15	1.10E-07	0.036	0.22	Component of the large ribosomal subunit. Participates in viral RNA transcription.
	TRIM8	E3 ubiquitin-protein ligase TRIM8	0.22	7.10E-14	0.26	2.20E-16	0.13	5.10E-06	Participates in multiple biological processes including cell survival, differentiation, apoptosis, and in particular, the innate immune response.TRIM8 as a mediator of IFN-γ responsiveness and macrophage activation syndrome (32).
	RPL35	60S ribosomal protein L35	−0.17	5.70E-09	0.17	5.00E-09	0.057	0.052	Component of the large ribosomal subunit. Participates in viral RNA transcription.
	MKNK2	MAP kinase-interacting serine/threonine-protein kinase 2	0.13	5.00E-06	0.17	5.10E-09	0.011	0.72	Serine/threonine-protein kinase that phosphorylates SFPQ/PSF, HNRNPA1, and EIF4E. MNK2 controls the macrophage antiinflammatory phenotype (33).
II	HINT1	Histidine triad nucleotide-binding protein 1	−0.12	5.40E-05	−0.1	5.80E-04	0.13	1.20E-05	Hydrolyzes purine nucleotide phosphoramidates with a single phosphate group. A potential marker of AD and Neuroplastic Mediator (34, 35). HINT1 regulates the interaction between mu-opioid receptors and NMDA receptors in the spinal cord and affects pain perception (36).
	FGF12	Fibroblast growth factor 12	−0.19	2.60E-10	−0.22	7.20E-15	−0.033	0.26	Involved in nervous system development and function. And the positive regulation of voltage-gated sodium channel activity. Promotes neuronal excitability and chemical synaptic release (37).
	MAPK9	Mitogen-activated protein kinase 9	−0.25	2.20E-16	−0.25	2.20E-16	−0.058	0.049	Serine/threonine-protein kinase is involved in various processes such as cell proliferation, differentiation, migration, transformation, and programmed cell death. Related to autophagy caused by Aβ monomer (38).
	ATP6V0B	V-type proton ATPase 21 kDa proteolipid subunit	−0.14	3.80E-06	−0.12	7.60E-05	0.1	3.80E-04	V-ATPase is responsible for acidifying a variety of intracellular compartments in eukaryotic cells. It is related to the acidification of autophagosomes.
	SLC6A15	Sodium-dependent neutral amino acid transporter B(0)AT2	−0.086	3.60E-03	−0.041	0.16	0.098	8.50E-04	Functions as a transporter for neurotransmitter precursors into neurons. Changes in SLC6A15 expression affect hippocampal neurochemistry and behavior, particularly glutamate transmission (39).
	VPS35	Vacuolar protein sorting-associated protein 35	−0.032	0.29	−0.12	4.60E-05	0.048	0.11	Acts as a component of the retromer cargo-selective complex to prevent missorting of selected transmembrane cargo proteins into the lysosomal degradation pathway. VPS35 is a key factor in tau phosphorylation and endosomal trafficking (40, 41). VPS35 is involved in the terminal differentiation of neurons, and its defects are risk factors for neurodegenerative diseases (42).
	SYN2	Synapsin-2	−0.17	5.80E-09	−0.19	2.70E-11	0.04	0.18	Neuronal phosphoprotein that coats synaptic vesicles, binds to the cytoskeleton, and is believed to function in the regulation of neurotransmitter release.
	ATP6V1C1	V-type proton ATPase subunit C 1	−0.18	1.10E-09	−0.23	1.50E-15	−0.053	0.071	V-ATPase is responsible for acidifying a variety of intracellular compartments in eukaryotic cells. It is related to the acidification of the autophagosome.
	RAB6B	Ras-related protein Rab-6B	−0.22	2.30E-14	−0.25	2.20E-16	−0.046	0.12	May function in intra-Golgi vesicle-mediated transport and retrograde transport of neuronal cells.
	ARPC5L	Actin-related protein 2/3 complex subunit 5-like protein	−0.28	2.20E-16	−0.29	2.20E-16	−0.053	0.074	May function as a component of the Arp2/3 complex, which is involved in the regulation of actin polymerization and mediates the formation of branched actin networks.
III	GAB1	GRB2-associated-binding protein 1	0.23	6.60E-15	0.26	2.20E-16	0.12	4.80E-05	Adapter protein plays a role in intracellular signaling cascades triggered by activated receptor-type kinases. Positive regulation of angiogenesis and oligodendrocyte differentiation (43).
	SALL1	Sal-like protein 1	0.15	4.20E-07	0.19	8.40E-11	0.11	1.80E-04	Transcriptional repressor involved in organogenesis. SALL1 is a microglia-specific gene and regulates microglia phenotype (44).
	PHF10	PHD finger protein 10	0.21	2.00E-13	0.23	5.40E-15	0.12	7.20E-05	Involved in transcription activity regulation by chromatin remodeling. PHF10 is necessary for neural progenitor cells to proliferate, differentiate into neurons after mitosis, and regulate dendritic growth.
	SPOCK3	Testican-3	0.063	0.034	0.12	4.30E-05	0.03	0.31	May participate in diverse steps of neurogenesis. It may affect the brain's fusiform gyrus (FUS) role in facial recognition (45). It could be genetic factors associated with delayed recall (46). Is an NIBP gene associated with severe developmental delay, corpus callosum dysplasia, and facial malformation(47).
	KRCC1	Lysine-rich coiled-coil protein 1	0.2	7.60E-12	0.2	1.30E-12	0.073	0.014	Also known as Cryptogenic hepatitis-binding protein 2.
	PAWR	PRKC apoptosis WT1 regulator protein	0.2	3.50E-12	0.2	3.70E-12	0.081	5.90E-03	Pro-apoptotic protein capable of selectively inducing apoptosis in cancer cells. Seems also to be a transcriptional repressor by itself. May be directly involved in regulating the amyloid precursor protein (APP) cleavage activity of BACE1.
	S100PBP	S100P-binding protein	0.13	1.70E-05	0.13	8.20E-06	0.095	1.20E-03	It may play a role in early tumorigenesis of pancreatic duct adenocarcinoma (48).
	CPM	Carboxypeptidase M	0.24	2.80E-16	0.3	2.20E-16	0.082	5.30E-03	Specifically removes C-terminal basic residues (Arg or Lys) from peptides and proteins. It is believed to play important roles in the control of peptide hormone and growth factor activity at the cell surface, and in the membrane-localized degradation of extracellular proteins. A marker of macrophage maturation (49).
	MYLIP	E3 ubiquitin-protein ligase MYLIP	0.14	1.50E-06	0.14	8.50E-07	0.14	1.20E-06	Inhibits neurite growth and low-density lipoprotein particle clearance. To participate in the neuron VLDLR regulation (50).
	BRD7	Bromodomain-containing protein 7	0.21	2.50E-13	0.23	2.30E-15	0.13	1.20E-05	It may play a role in chromatin remodeling. Promotes oligodendrocyte differentiation and myelination (51). Plays an anti-inflammatory role in early acute inflammation(52).

### Validation of diagnostic value

**Figure 11:**
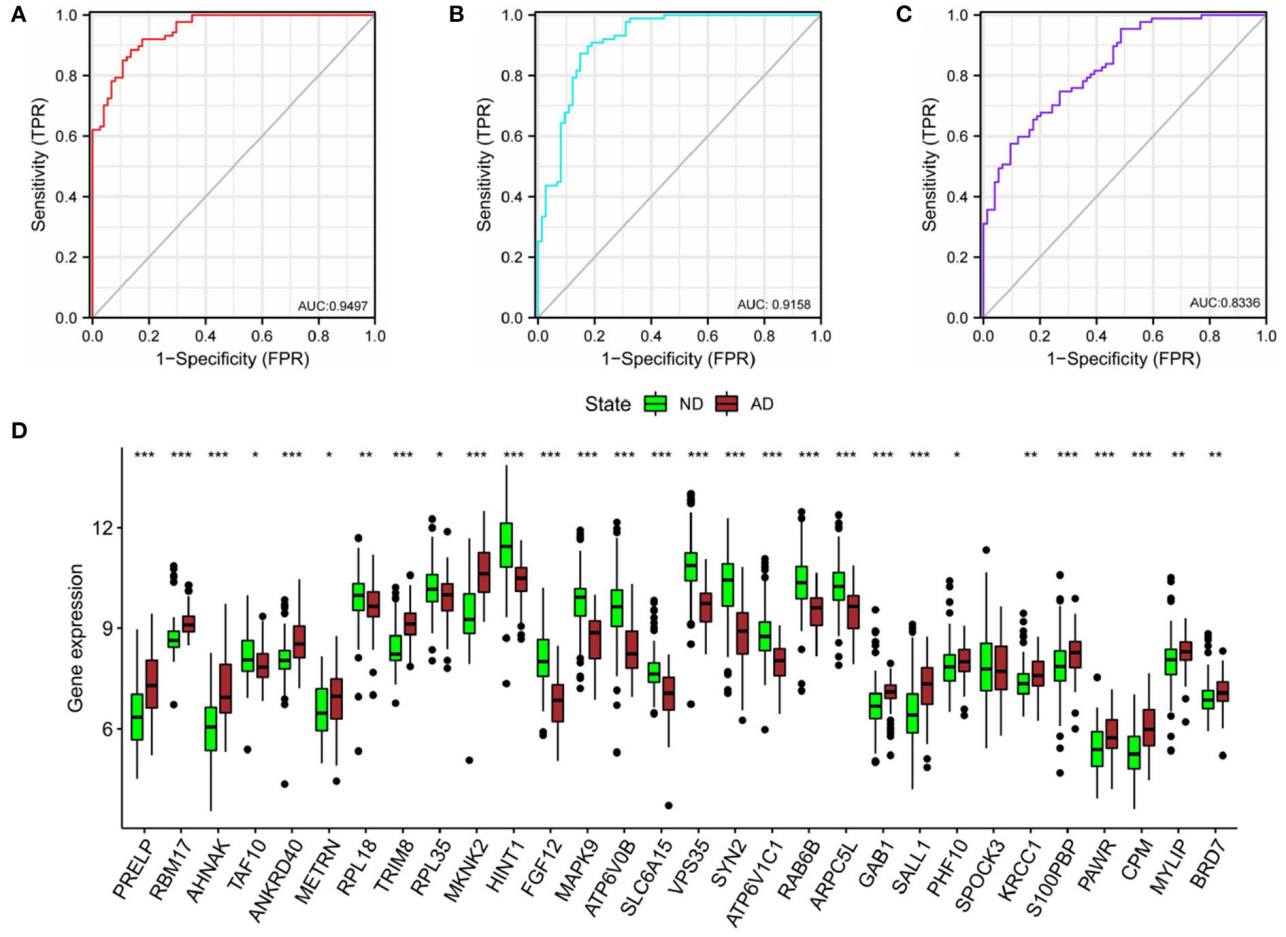
Validation of diagnostic value. **(A)** The combined diagnostic value of eight genes in subgroup I. **(B)** The combined diagnostic value of eight genes in subgroup II. **(C)** The combined diagnostic value of eight genes in subgroup III. glm function to build logistics model, pROC package for ROC analysis. **(D)** Expression of hub genes in ND and AD groups. *n* = 74–87. vs. ND, Wilcoxon's rank-sum test, *P < 0.05; **P < 0.01; ***P < 0.001.

In order to verify the diagnostic values of top10 genes in each subgroup, one new dataset, GSE5281 (19–22) was used. We tested diagnostic values for single-gene and combination-gene in each subgroup; a total of 3,069 options were offered (Supplementary File 7). The combination of eight genes is the optimal molecular marker diagnostic scheme. AUC was 0.950, 0.916, 0.834 in subgroup I, subgroup II, and subgroup III, respectively (Table 2, Figures 11A–C). Generally, with an AUC of 0.7–0.9, there is a diagnostic value, and the diagnostic value was high when the AUC was above 0.9. Furthermore, the boxplot shows that the majority of hub genes (29/30) were different from the normal group (Figure 11D). All these proofs indicated that a combination of eight marker genes could predictive of AD. It provides a valuable model for the development of diagnostic chips.

**Table 2:** Subgroup best combined diagnosis genes.

**Subgroup**	**gene1**	**gene2**	**gene3**	**gene4**	**gene5**	**gene6**	**gene7**	**gene8**	**AUC**
I	PRELP	AHNAK	TAF10	ANKRD40	METRN	RPL18	TRIM8	MKNK2	0.9497
II	HINT1	FGF12	ATP6V0B	SLC6A15	VPS35	SYN2	ATP6V1C1	RAB6B	0.9158
III	SALL1	PHF10	SPOCK3	KRCC1	S100PBP	CPM	MYLIP	BRD7	0.8336

## Discussion

### Implications of our research

Alzheimer's disease has attracted extensive attention and research worldwide. Unfortunately, so far, attempts to find drug interventions that can alter the onset or progression of dementia have failed (53). The continued failure is attributable to (I) extensive irreversible damage already present at the stage of clinical symptoms of the disease process; (II) lack of precise intervention targets in multifactorial conditions (8). Therefore, scientists are committed to finding multimodal biomarkers to find high-risk groups in the preclinical asymptomatic stage and distinguish patients according to their pathophysiological status to achieve precise intervention. The bygone research paradigm was from clinical phenotype to molecular phenotype, while genomics was the opposite. Genomic subtype analysis of multi-sample multi-gene status can help us capture genetic heterogeneity in AD patients. Interestingly, Frisoni et al. (54) also believed that Alzheimer's disease was far from a single disease with the same cause and the same impact. The analysis suggested that patients be divided into three groups, each with its own dynamic changes. However, AD genomic subtypes were blank to our knowledge, and our research filled this gap.

**Figure 12:**
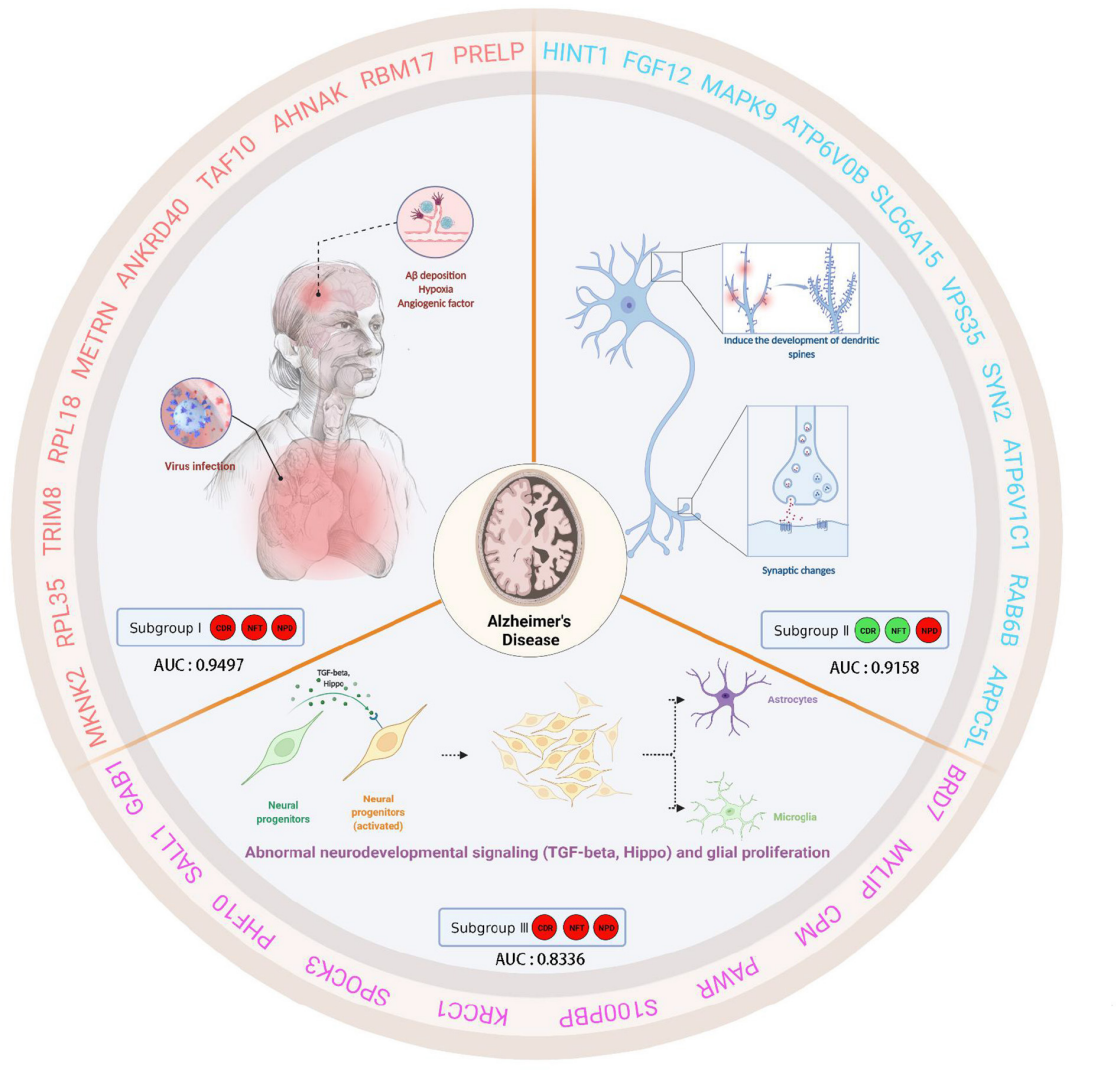
This schematic diagram describes the clinical characteristics, biological significance, and hub genes of three subgroups of AD. Subgroup I showed high CDR, NFT, and NPD, which were closely related to virus infection. Furthermore, subgroup I showed characteristic genes associated with hypoxia factors and angiogenesis factors. Subgroup II, low CDR, NFT, and high NPD, had minor synaptic damage. In subgroup II, spinous dendritic development and synaptic plasticity were upregulated, axonal vesicle transport and synaptic transmission were upregulated, autophagy and phagosome acidification were increased, and postsynaptic membrane transmitter receptors (especially NMDA receptors and AMPA receptors associated with long-term potentiation, post-scaffold protein PSD95) were upregulated, and intracellular signaling was active. Subgroup III presented high CDR, NFT, and NPD, which was similar to familial AD due to PSEN1-specific amplification. Subgroup III showed TGF-beta and active Hippo signaling pathway, self-renewal of neural stem cells, the proliferation of neural progenitor cells, differentiation and activation of glial cells, etc. We speculated that these changes in subgroup III were cross-talk.

This study analyzed gene expression profiles of AD cases and ND people from five independent GEO datasets. We can find that the batch effects from different platforms or batches were successfully removed. In addition, 743 AD brain tissue samples were robustly classified into three subgroups based on gene expression profiles for the first time. Moreover, the three subgroups were evaluated according to the “A/T/N” system. Transcriptome classification reviews subgroup-specific functions and pathways, explaining the pathological characteristics of each subgroup. Further, we identified the core genes and confirmed the correlation between the core genes and clinical features. Finally, we verified that the core gene has good diagnostic value. The schematic diagram is shown in Figure 12 which was created with BioRender.com.

### Clinical features of subtypes

Clinical annotation makes subgroups more strongly associated with clinical variables than unsupervised subtypes based solely on genetic profile similarity. In this section, we drew three momentous conclusions:

A. Subgroups I and III had a higher risk of cognitive decline than subgroup II. We found that the three subgroups had almost the same severe amyloid plaque load. However, the degree of clinical dementia and NFT in subgroups I and III were higher than those in subgroup II. There was no significant difference between subgroups I and III.
B. Furthermore, we found that the trends of the three-course labels were not completely parallel. In our research, there was a correlation between NFT and clinical dementia score (*r* = 0.39), significant in identifying the elderly at risk of cognitive decline. In contrast, amyloid plaque deposition did not correlate with clinical dementia scores. The clinical study conducted by Dumugier et al. (55) also reached a similar conclusion. Baseline CSF t-tau, p-tau, and hippocampal volume were independently related to the decline of future cognitive ability. Frisoni et al. (54) also consider supporting evidence for inconsistencies in the current conceptualization of the amyloid hypothesis. Tau protein, a marker of neuronal injury, combined with mental symptoms, can be used as a standard for the severity classification of AD (3).
C. High amyloid plaques were found in all three subgroups, suggesting that Aβ deposition is a corporate pathogenic mechanism in AD patients. CSF t-Tau, P-Tau, and neurogranuloprotein increased only in Aβ -positive individuals during the entire aging process (56). Cognition decreases were measurable at subthreshold levels of Aβ deposition (6). This prompts us to reconsider whether amyloid plaques are at the core of AD pathology or a key target for clinical outcomes. Drugs targeting tau pathology may be more effective in improving cognition.

### Biological function of subgroups

#### Subgroup I

Subgroup I is mainly involved in the cotranslation protein of targeted membrane, viral gene expression, vascular endothelial growth factor signaling pathway. Pathway analysis involved ribosome, HIF-1 signaling pathway. Such results attract our interest in the association between subgroup I patients and viral infection, hypoxia injury, and angiogenesis.

Infection promotes amyloid deposition and neuroinflammatory pathology in the brain. Human herpes virus, cytomegalovirus, and hepatitis C virus may be pathogenic factors of AD (57). Our findings demonstrated a strong association between subgroup I and viral infection, confirming the viral theory in AD pathogenesis.

Subgroup I was also covered in vascular endothelial factors caused by hypoperfusion hypoxia, such as hypoxia-inducible factor HIF-1, vascular endothelial growth factor, and Notch signaling. Hypoxia-induced vascular growth factors are known to accumulate in the brain of AD patients, especially near Aβ plaques; however, due to the imbalance of lateral inhibition of angiogenesis, the non-productive angiogenesis (NPA) pathway leads to the aggregation of abnormal vascular structures around Aß plaques (58).

#### Subgroup II

Under the same high amyloid plaque load, fibrous tangles and clinical dementia were the lightest in subgroup II. Specific genes reveal the heterogeneity of subgroup II in synaptic pathology. Since changes in synaptic function are associated with changes in synaptic protein concentration, several significant synaptic protein genes in these pathways are beneficial in the treatment of AD.

The differences in synaptic function in subgroup II can be explained in four directions: ① Inducing dendrite development, and synaptic plasticity (DLGAP1, PSD-95, SHANK2, HINT1 PAK1, PAK3, EPHA4). ② Axonal vesicle transport and synaptic transmission are upregulated (SNCA, PAK1, SYN2, VPS35, CACNA1B, RIMS1). ③ Increased axon terminal autophagy and increased phagosome acidification (MAPK9, MAPK10, ATP6V0B, ATP6V1C1, SNCA). ④ Postsynaptic membrane transmitter receptors are upregulated, and intracellular signal transmission is active (GRIN2A, GRIA3, GRIA4, GABRA1, HTR2A, GNB5, ADCY1, KCNJ3).

Synaptic fluctuations precede neuronal changes and are directly associated with cognitive deficits in the early stages of dementia (59). Synaptic proteins may be biological targets closer to disease specificity, and treatment based on synaptic repair and regeneration can play a role in the early stages of lesions. We suspect subgroup II synaptic proteins change a compensatory protection factor; dementia early triggers these protective factors against the decline of cognitive function.

#### Subgroup III

Subgroup III is considered to be the most dangerous subtype. PSEN1 is an essential gene specifically upregulated in subgroup III, although gene amplification is not entirely due to gene mutation. CpG alters in methylation patterns are also a factor (60). Group III is still considered a suspected familial AD phenotype.

The pathophysiological features typical of subgroup III can be described as abnormal neurogenic signaling (including cell adhesion, TGF-beta, Hippo, SMAD, stem cell pluripotency, etc.) and glial proliferation. Previous studies have shown that neurodevelopment and degeneration coexist in AD. GO analysis indicated that subgroup III might participate in the self-renewal of neural stem cells, the proliferation of neural progenitor cells, differentiation and activation of glial cells, and myelination of glial cells through the Hippo pathway (61, 62). Transforming growth factor TGF-beta is a downstream signaling molecule of the Hippo pathway. It is continuously expressed in adult microglia as a critical regulator of glial differentiation and function (63, 64).

The findings of subgroup III suggest the double-edged influence of neural stem cell biology and glial cells in the pathogenesis of AD, and whether the effect is related to presenilin mutation needs to be verified.

### Hub gene screening and subtype validation

The repeatability of subtypes is an important index to detect and evaluate effectiveness. Experiments that lacked out-of-sample validation tended to report near-perfect areas under the curve (AUC), while papers that performed out-of-sample cross-validation reported milder and more convincing results (8). We took the TOP10 genes of GSEA in each subgroup as the core genes and introduced an additional independent group sample for model evaluation.

We found that core genes in subgroup I and subgroup III were positively correlated with the “A/T/N” system, while those in subgroup II were negatively correlated. Furthermore, the radar map shows similar results. The functional interpretation of hub genes of subgroups shows in Table 1. Comprehensive functional analysis of subgroups, we speculate subgroup I and subgroup III were high-risk subgroups of AD. It also may represent a “pure AD” population, while subgroup II may reflect AD-like dementia.

In the validation of hub genes by external data sets, the boxplot of differential genes showed the same results. Passing the 3,069 measurements, we discover that the united diagnosis of eight genes had the maximum worth. This may provide a regular reference value for the accurate diagnosis of AD. Note, the concept of AD molecular subtypes can not only describe the clinical and pathological heterogeneity but also distinguish it from other age-related dementia and normal aging. The set of AD molecular subtypes reported in this study has the potential to be developed for microarray detection in clinical blood samples.

### Deficiencies and prospects

Although the study combined multiple unsupervised subtype maps into biological subtypes with clinical predictive value through further analysis, subtypes in the animal model test are tough to carry out. Like any single omics approach, data-driven biomarkers do not directly consider the multi-gene and multi-factor mechanisms that influence AD. Current phenotypic and omics studies focus on association analysis of genome and disease rather than causal relationship exploration. From analysis of only the pathogenic portion, some key information may be hidden. Exploration after intervention is required (65).

Further research is needed to expand the scope of finding gene-gene or gene-environment phenotypic-phenotypic interactions, thereby building a multiscale, layered, dynamic framework for AD brain network research and translating it into clinical practice (9). Reproducibility analysis of such systems will be a potential area of future work.

## Conclusion

Precise and early diagnosis of AD is a problem encountered worldwide. Diagnostic markers of AD were found based on transcriptomics. The diagnostic model constructed by combining eight genes showed excellent diagnostic value. This provides a research basis for early and accurate diagnosis of AD.

## Data availability statement

The original contributions presented in the study are included in the article/Supplementary material, further inquiries can be directed to the corresponding author.

## Ethics statement

Ethical review and approval was not required for the study on human participants in accordance with the local legislation and institutional requirements. Written informed consent from the patients/ participants or patients/participants' legal guardian/next of kin was not required to participate in this study in accordance with the national legislation and the institutional requirements.

## Author contributions

XC designed the study and modified the manuscript. DQ and TY reviewed the data. HL and MW carried the specific studies and wrote the article. YL searched the database literature. All authors contributed to the article and approved the submitted version.

## Funding

The Major basic research projects of the Shandong Natural Science Foundation (ZR2020ZD17), Natural Science Foundation of Shandong Province (ZR2021QH157) and Key R&D program of the science and technology program of Tibet Autonomous Region (XZ202201ZY0026G) financially supported this research work. Dr. Wenchao Li [Department of Computational Biology for Individualized Infection Medicine, Centre for Individualized Infection Medicine (CiiM), a joint venture between the Helmholtz-Centre for Infection Research (HZI) and the Hannover Medical School] assisted part of the bioinformatics analysis.

## Conflict of interest

The authors declare that the research was conducted in the absence of any commercial or financial relationships that could be construed as a potential conflict of interest.

## Publisher's note

All claims expressed in this article are solely those of the authors and do not necessarily represent those of their affiliated organizations, or those of the publisher, the editors and the reviewers. Any product that may be evaluated in this article, or claim that may be made by its manufacturer, is not guaranteed or endorsed by the publisher.

## Supplementary material

Supplementary File 1 The clinical characteristics of each sample.

Supplementary File 2 Differential gene expression.

Supplementary File 3 AD subgroups.

Supplementary File 4 GO and KEGG details.

Supplementary File 5 Gene expression heatmap in each pathway.

Supplementary File 6 GSEA gene ranking.

Supplementary File 7 ROC.

Supplementary File 8 List of major acronyms.
