# Supplementary figures and images for: Identification of the molecular subgroups in Alzheimer's disease by transcriptomic data
Source: Front Neurol. 2022 Sep 20;13:901179. doi: 10.3389/fneur.2022.901179 (PMC9530954; doi:10.3389/fneur.2022.901179)

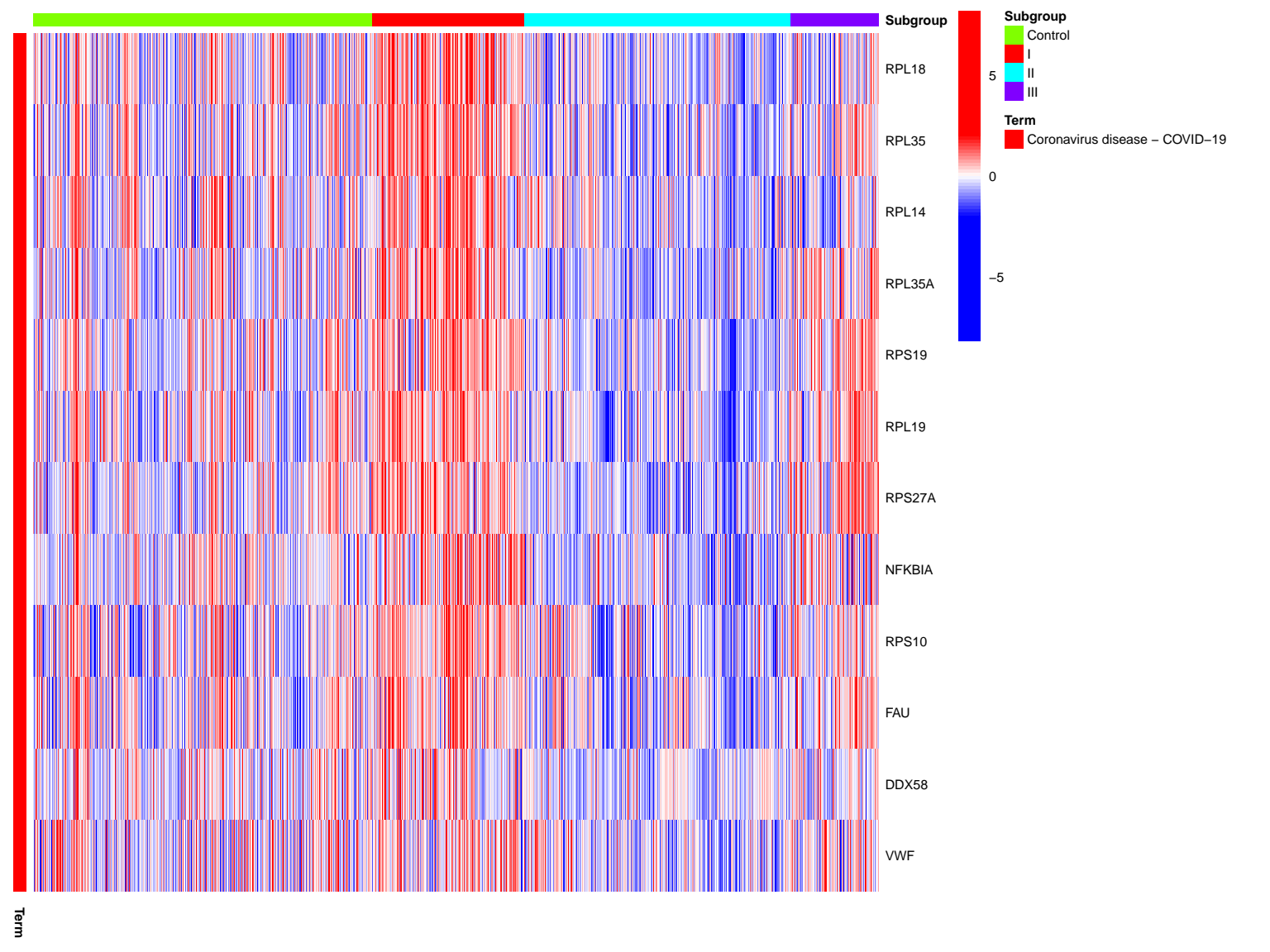

Supplement: Supplementary File 5 — Gene expression heatmap in each pathway. [file Data_Sheet_1.ZIP › Supplementary File 5.gene expression heatmap in each pathway/I-Coronavirus disease - COVID-19.pdf]

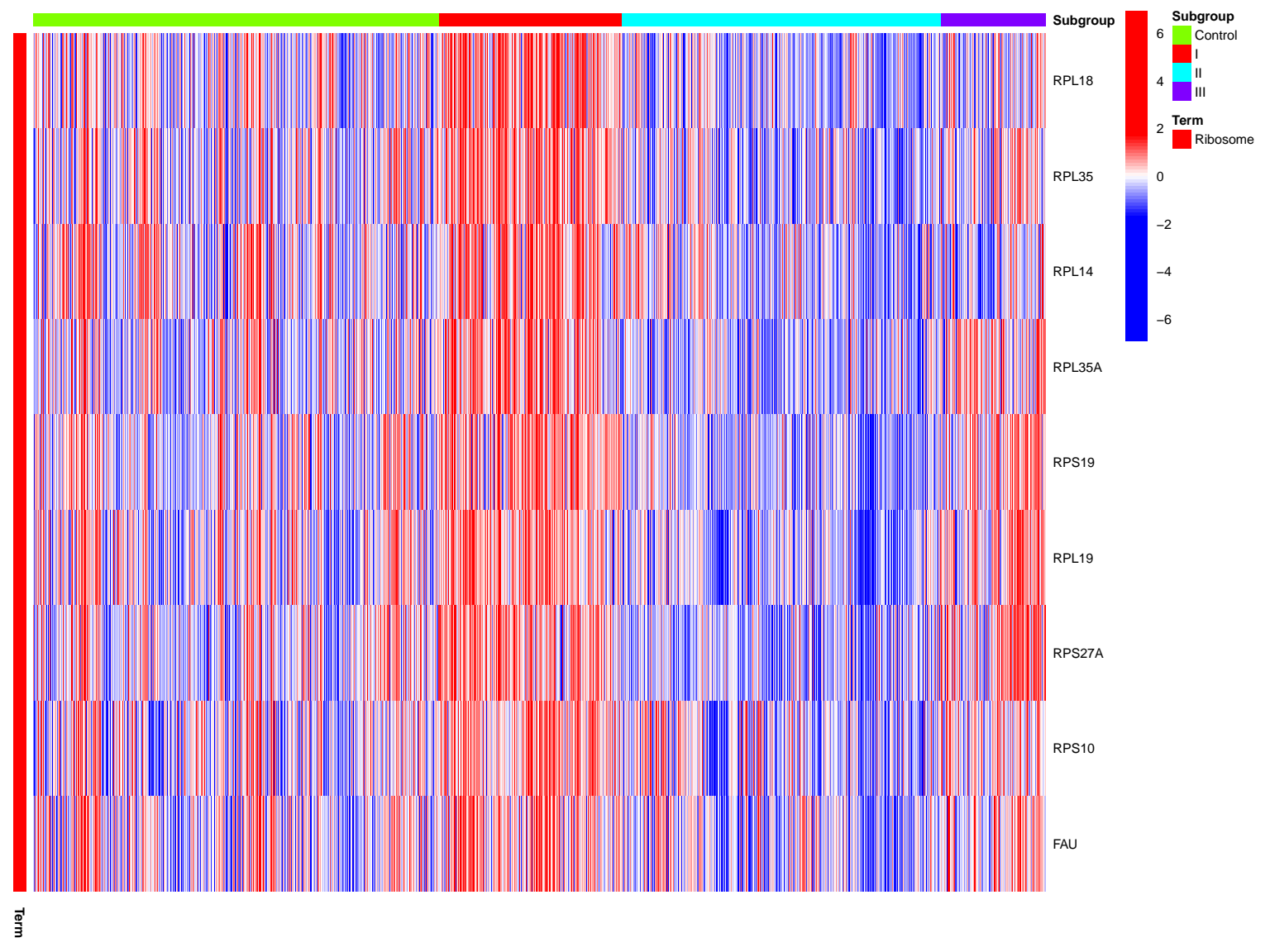

Supplement: Supplementary File 5 — Gene expression heatmap in each pathway. [file Data_Sheet_1.ZIP › Supplementary File 5.gene expression heatmap in each pathway/I-HIF-1 signaling pathway.pdf]

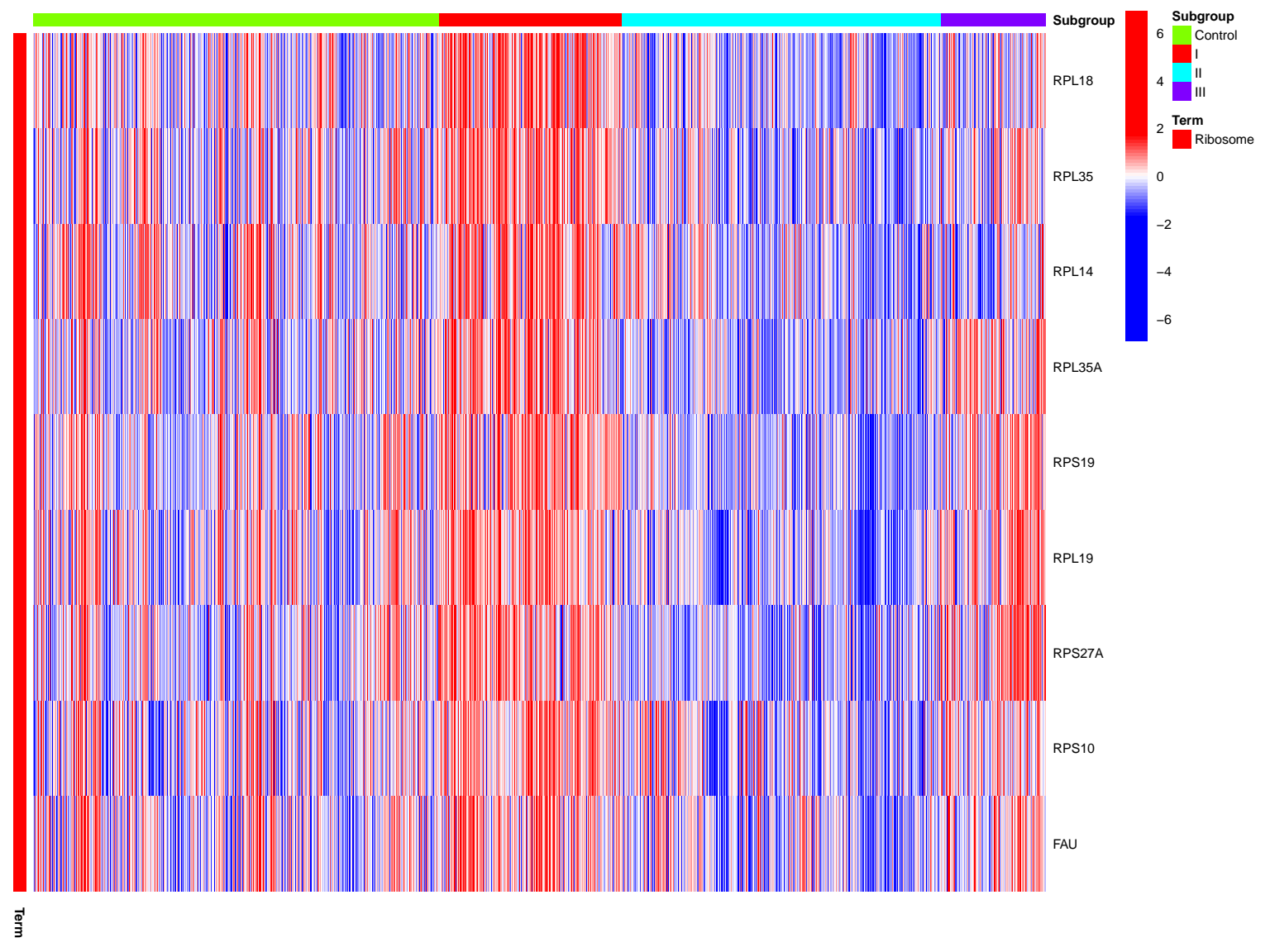

Supplement: Supplementary File 5 — Gene expression heatmap in each pathway. [file Data_Sheet_1.ZIP › Supplementary File 5.gene expression heatmap in each pathway/I-Ribosome.pdf]

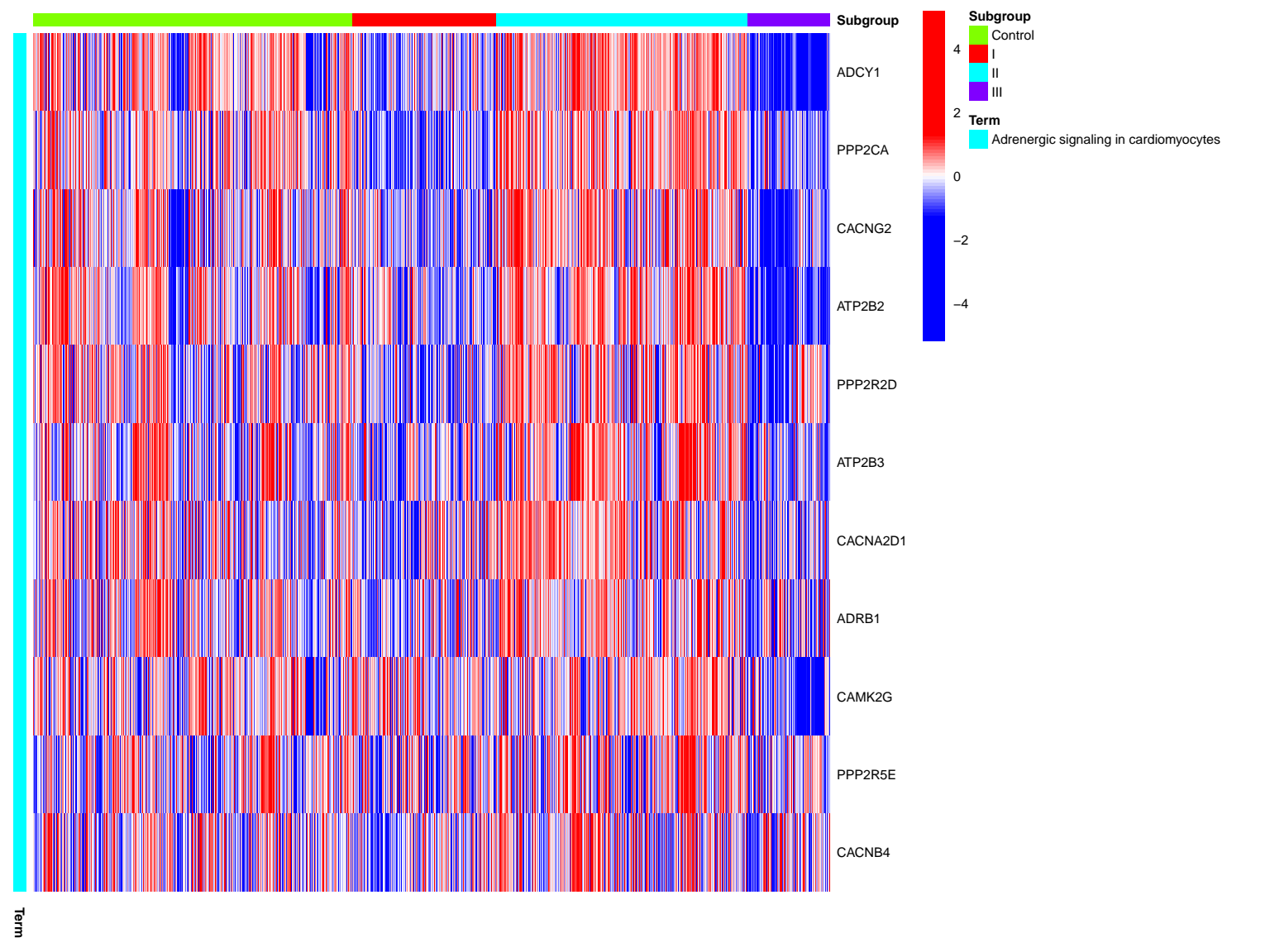

Supplement: Supplementary File 5 — Gene expression heatmap in each pathway. [file Data_Sheet_1.ZIP › Supplementary File 5.gene expression heatmap in each pathway/II-Adrenergic signaling in cardiomyocytes.pdf]

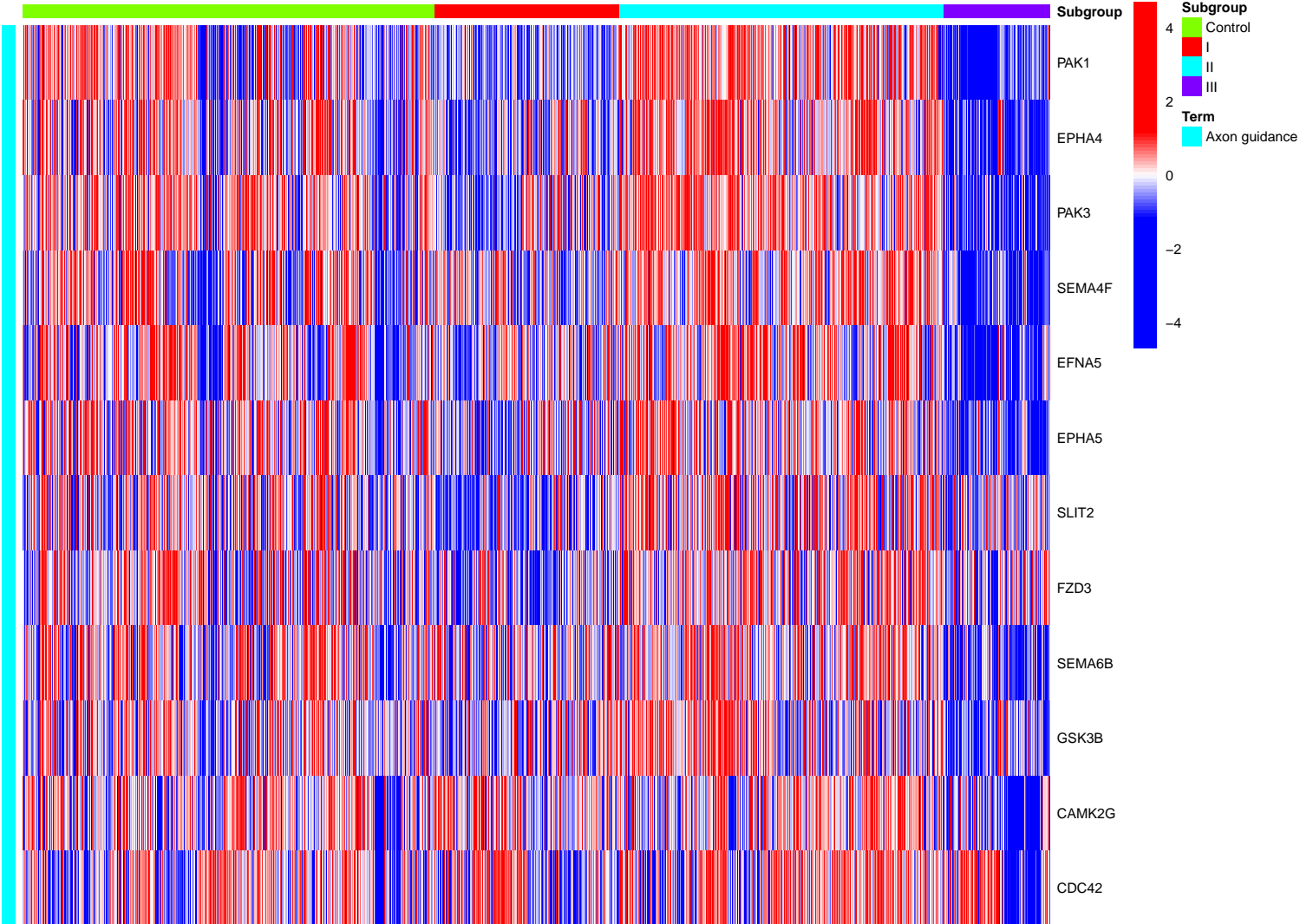

Term

Supplement: Supplementary File 5 — Gene expression heatmap in each pathway. [file Data_Sheet_1.ZIP › Supplementary File 5.gene expression heatmap in each pathway/II-Axon guidance.pdf]

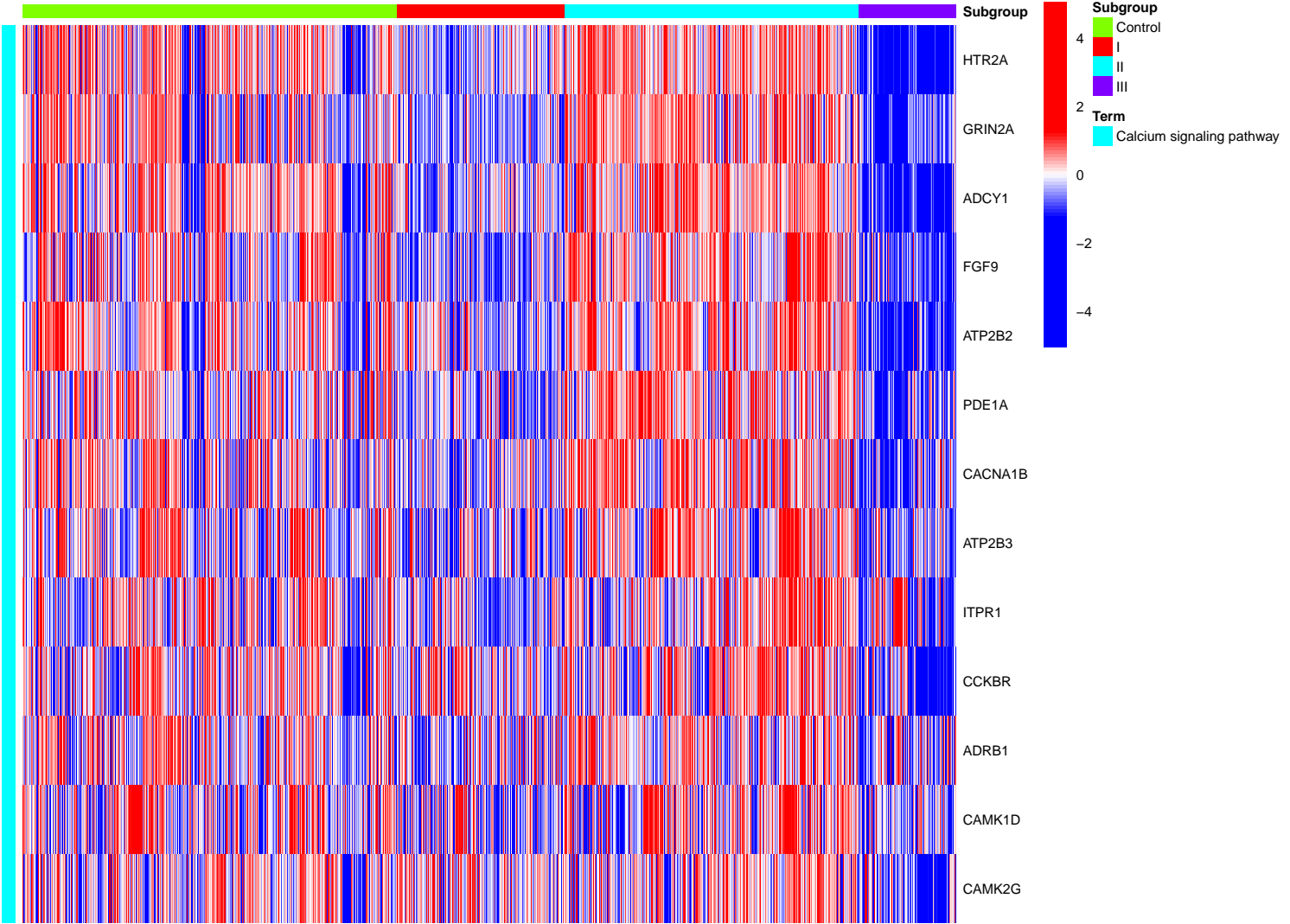

Term

Supplement: Supplementary File 5 — Gene expression heatmap in each pathway. [file Data_Sheet_1.ZIP › Supplementary File 5.gene expression heatmap in each pathway/II-Calcium signaling pathway.pdf]

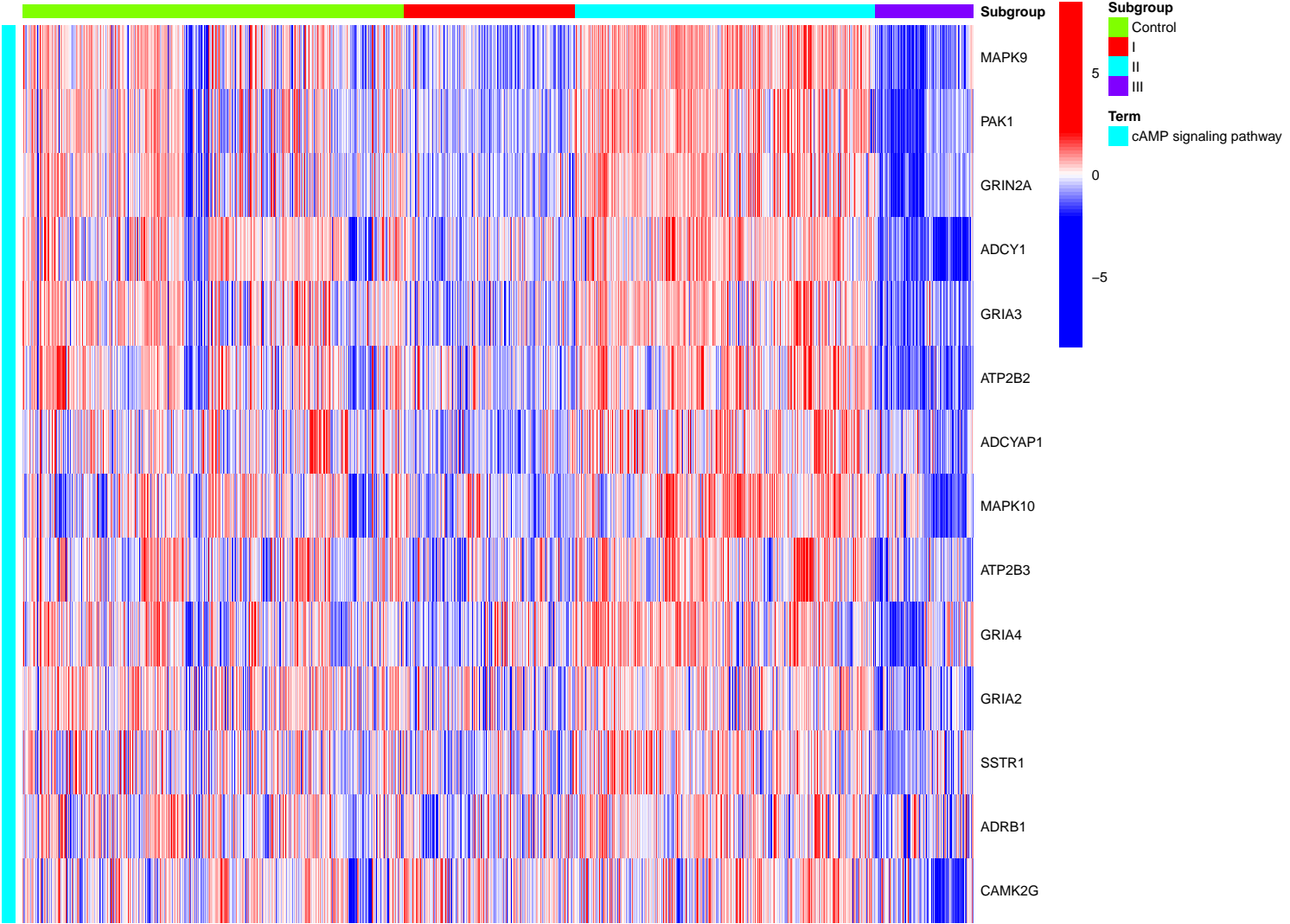

Term

Supplement: Supplementary File 5 — Gene expression heatmap in each pathway. [file Data_Sheet_1.ZIP › Supplementary File 5.gene expression heatmap in each pathway/II-cAMP signaling pathway.pdf]

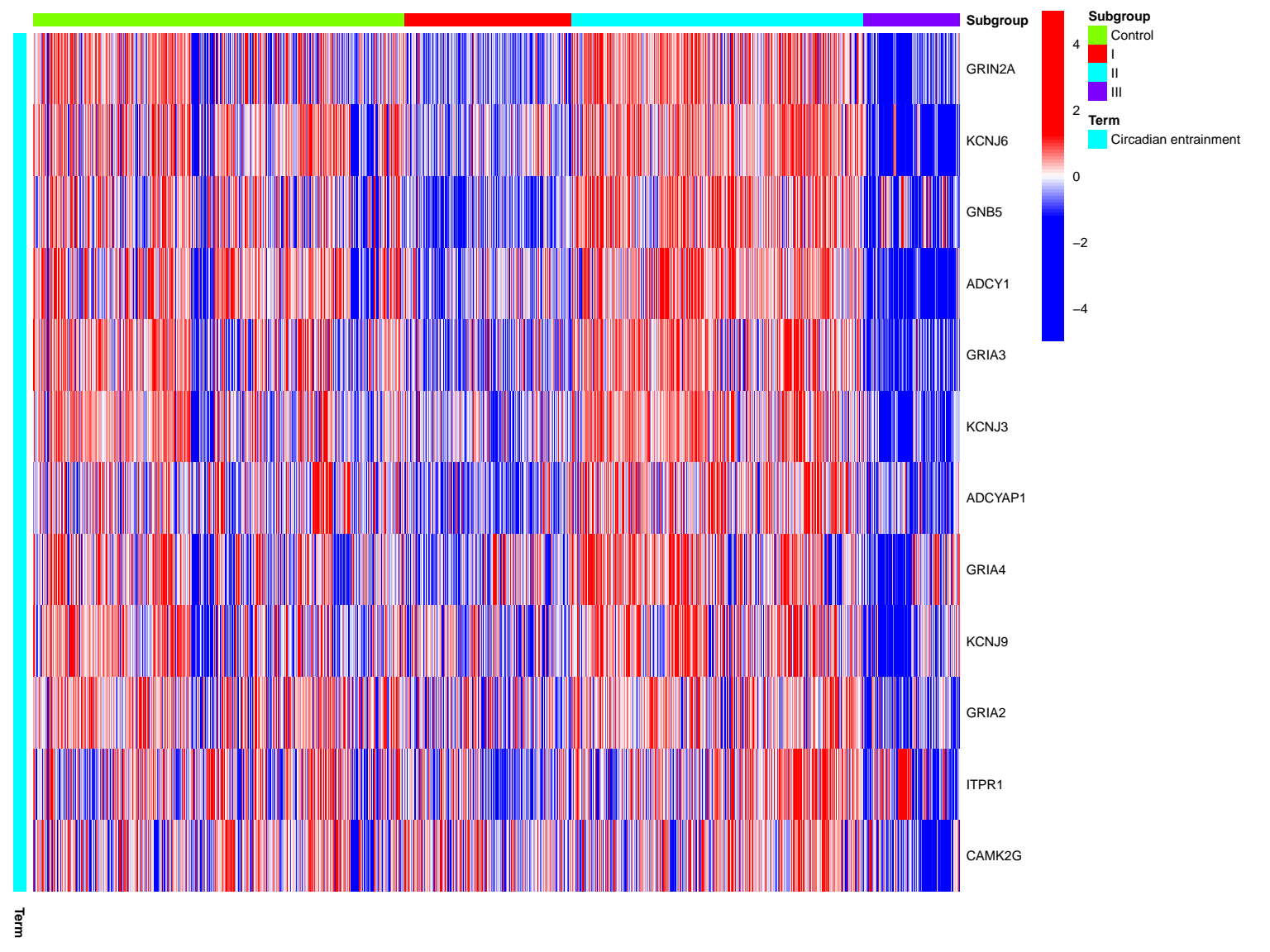

Supplement: Supplementary File 5 — Gene expression heatmap in each pathway. [file Data_Sheet_1.ZIP › Supplementary File 5.gene expression heatmap in each pathway/II-Circadian entrainment.pdf]

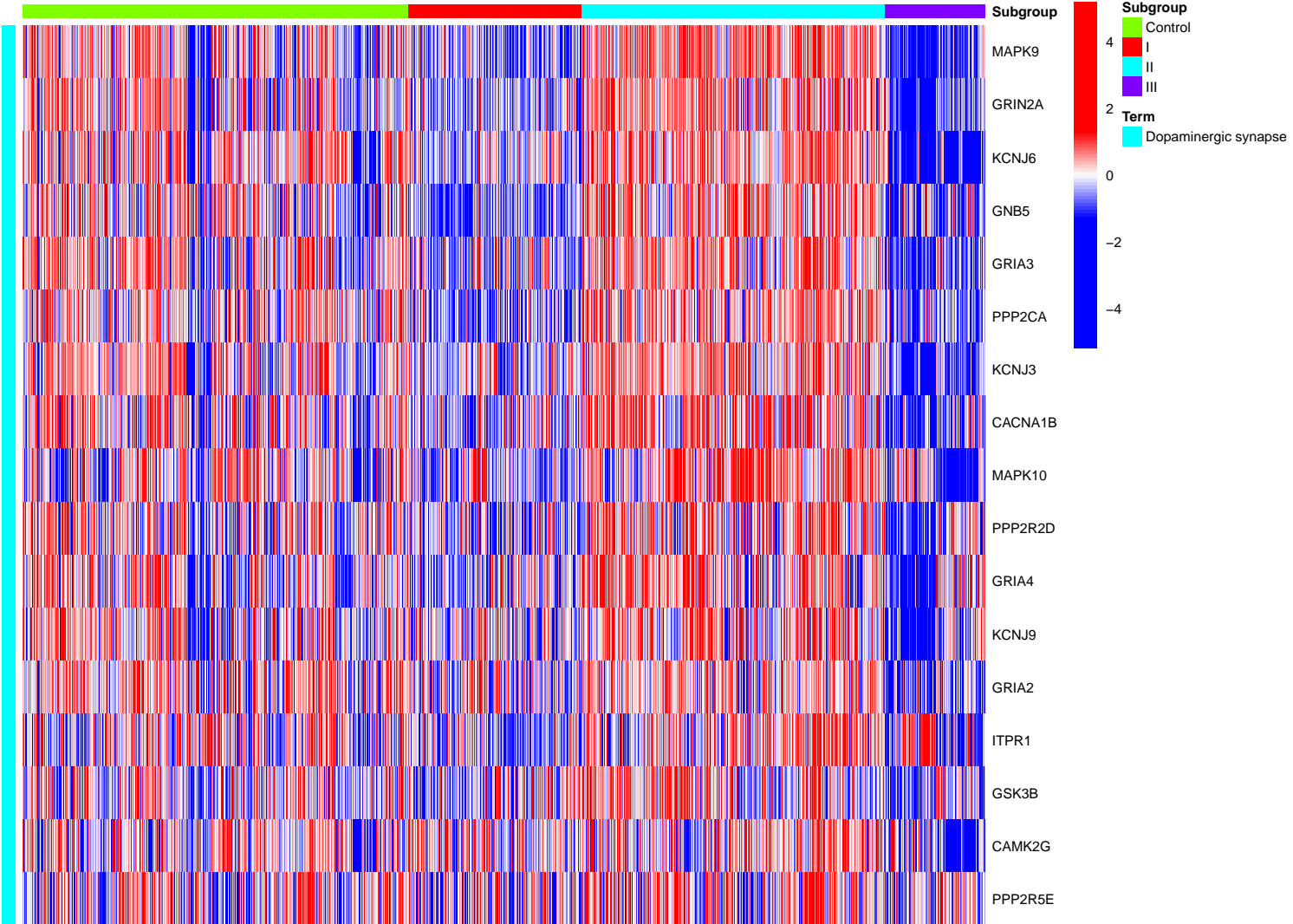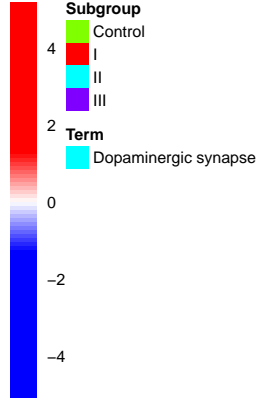

Term

Supplement: Supplementary File 5 — Gene expression heatmap in each pathway. [file Data_Sheet_1.ZIP › Supplementary File 5.gene expression heatmap in each pathway/II-Dopaminergic synapse.pdf]

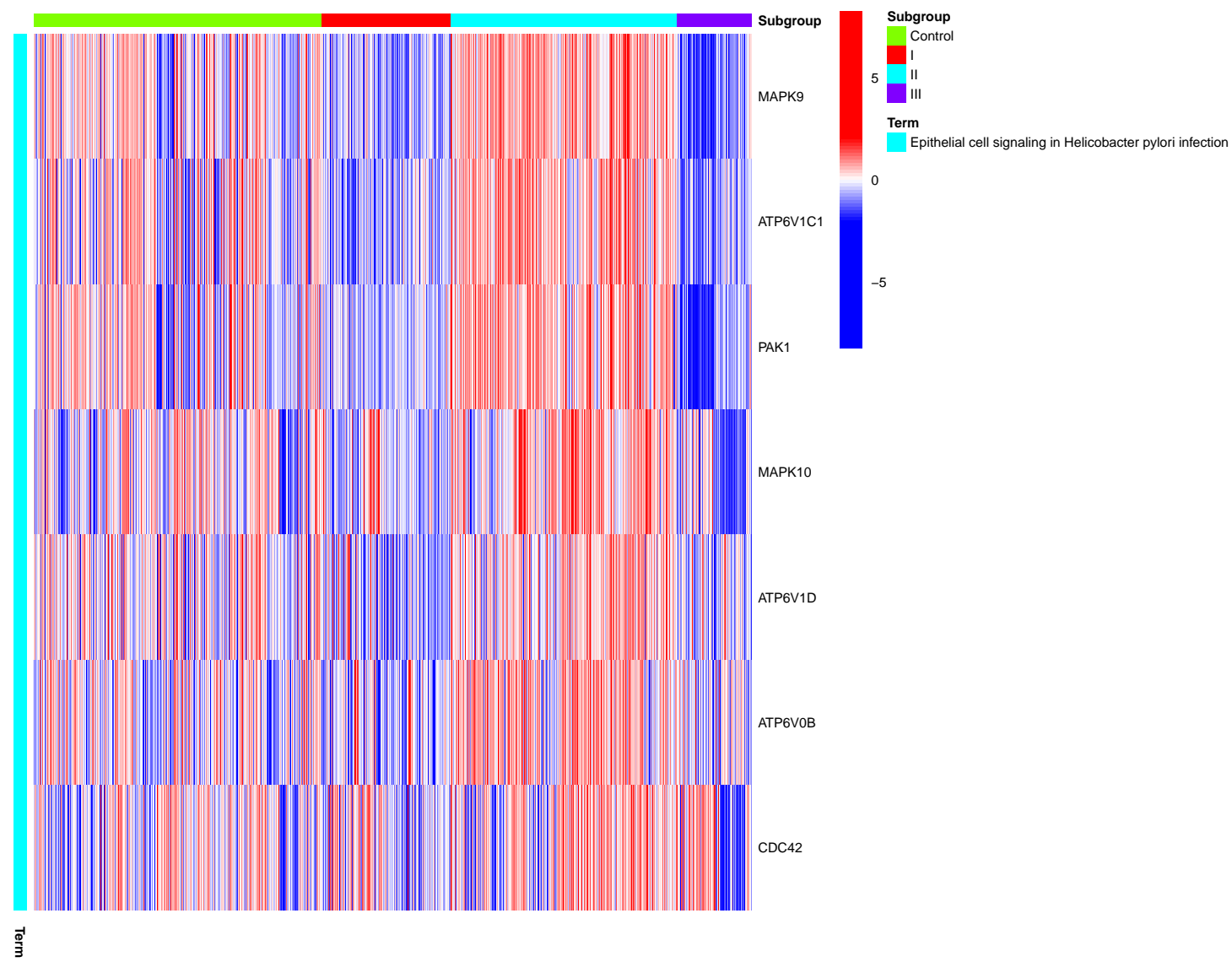

Supplement: Supplementary File 5 — Gene expression heatmap in each pathway. [file Data_Sheet_1.ZIP › Supplementary File 5.gene expression heatmap in each pathway/II-Epithelial cell signaling in Helicobacter pylori infection.pdf]

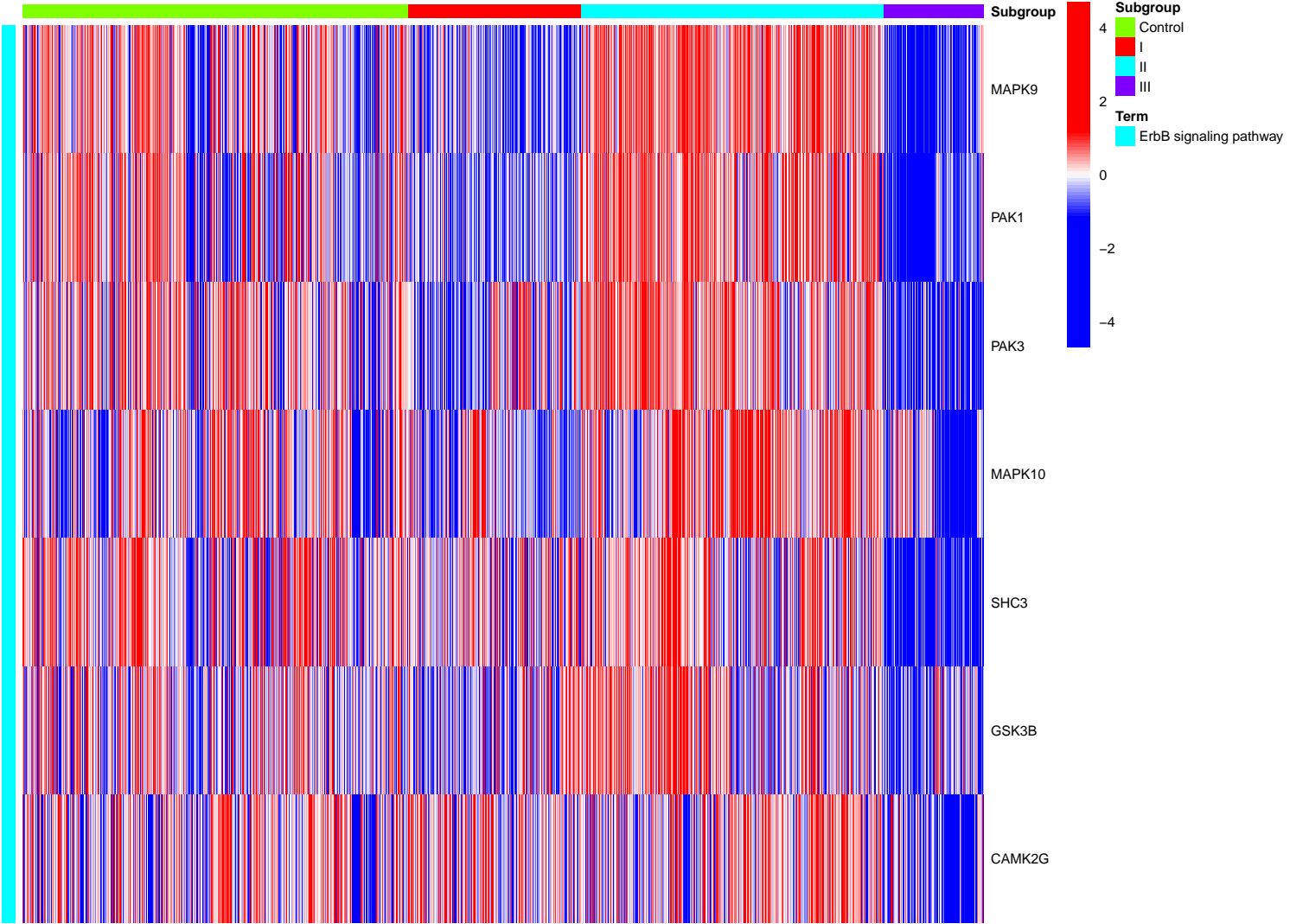

Term

Supplement: Supplementary File 5 — Gene expression heatmap in each pathway. [file Data_Sheet_1.ZIP › Supplementary File 5.gene expression heatmap in each pathway/II-ErbB signaling pathway.pdf]

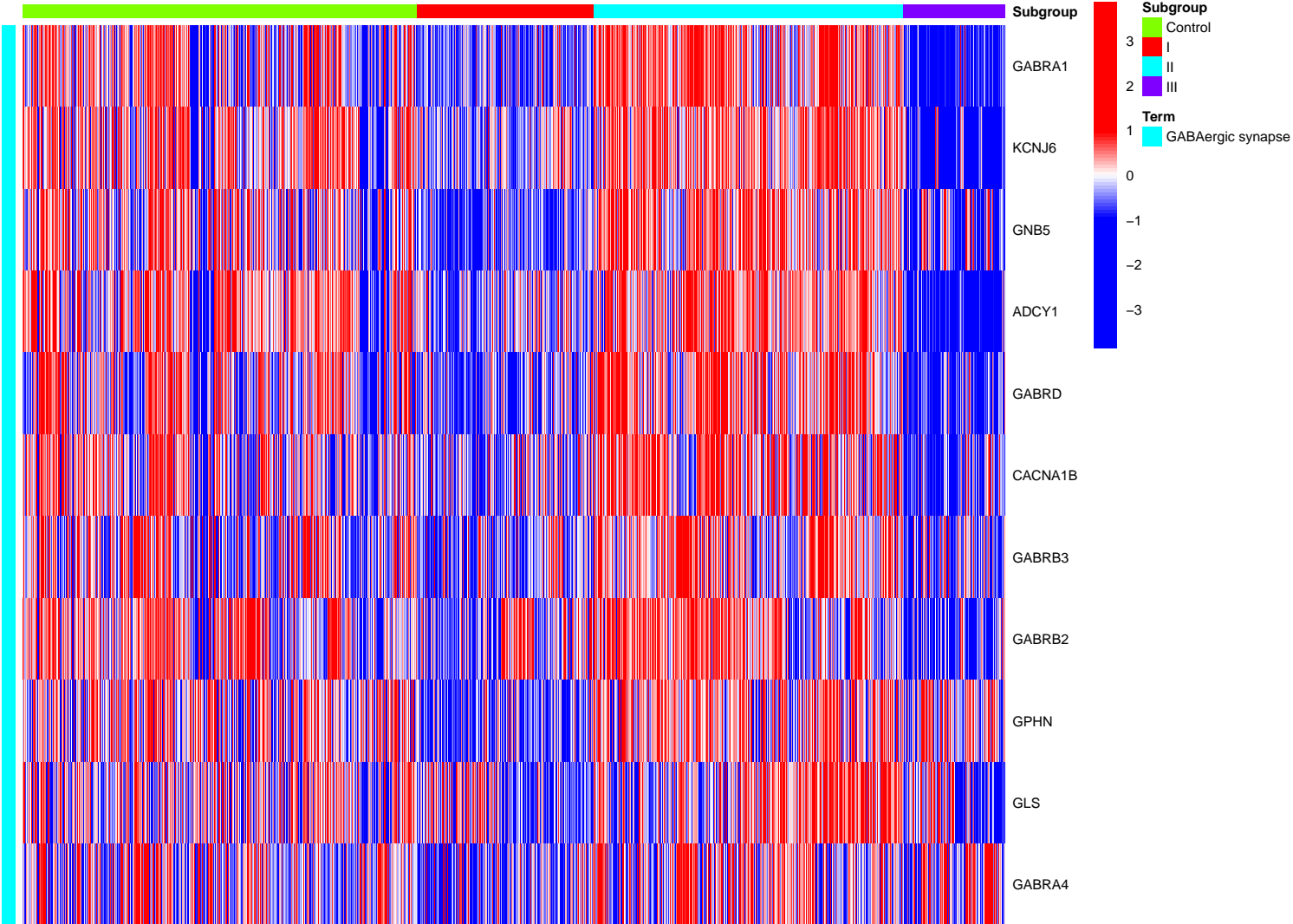

Term

Supplement: Supplementary File 5 — Gene expression heatmap in each pathway. [file Data_Sheet_1.ZIP › Supplementary File 5.gene expression heatmap in each pathway/II-GABAergic synapse.pdf]

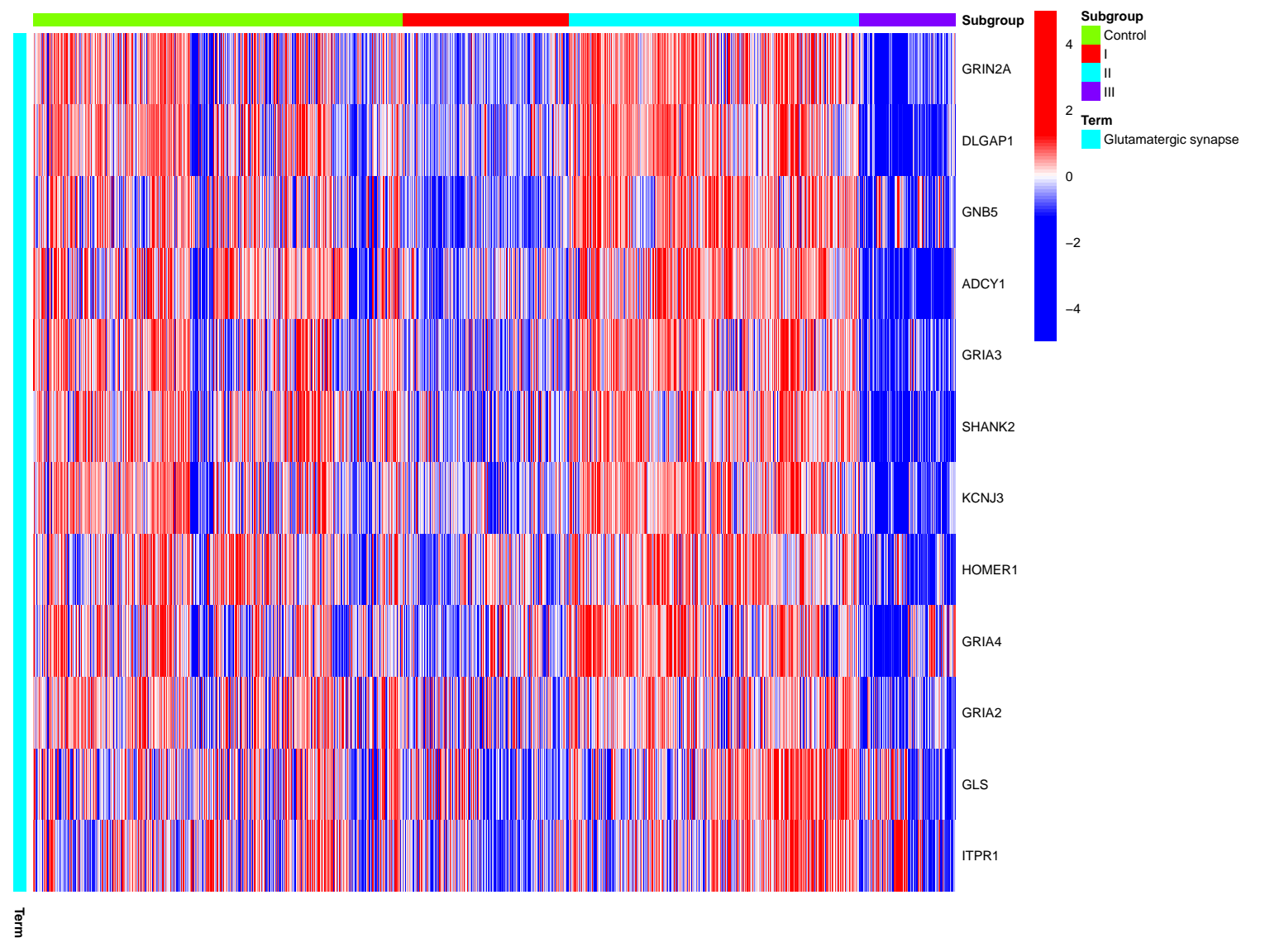

Supplement: Supplementary File 5 — Gene expression heatmap in each pathway. [file Data_Sheet_1.ZIP › Supplementary File 5.gene expression heatmap in each pathway/II-Glutamatergic synapse.pdf]

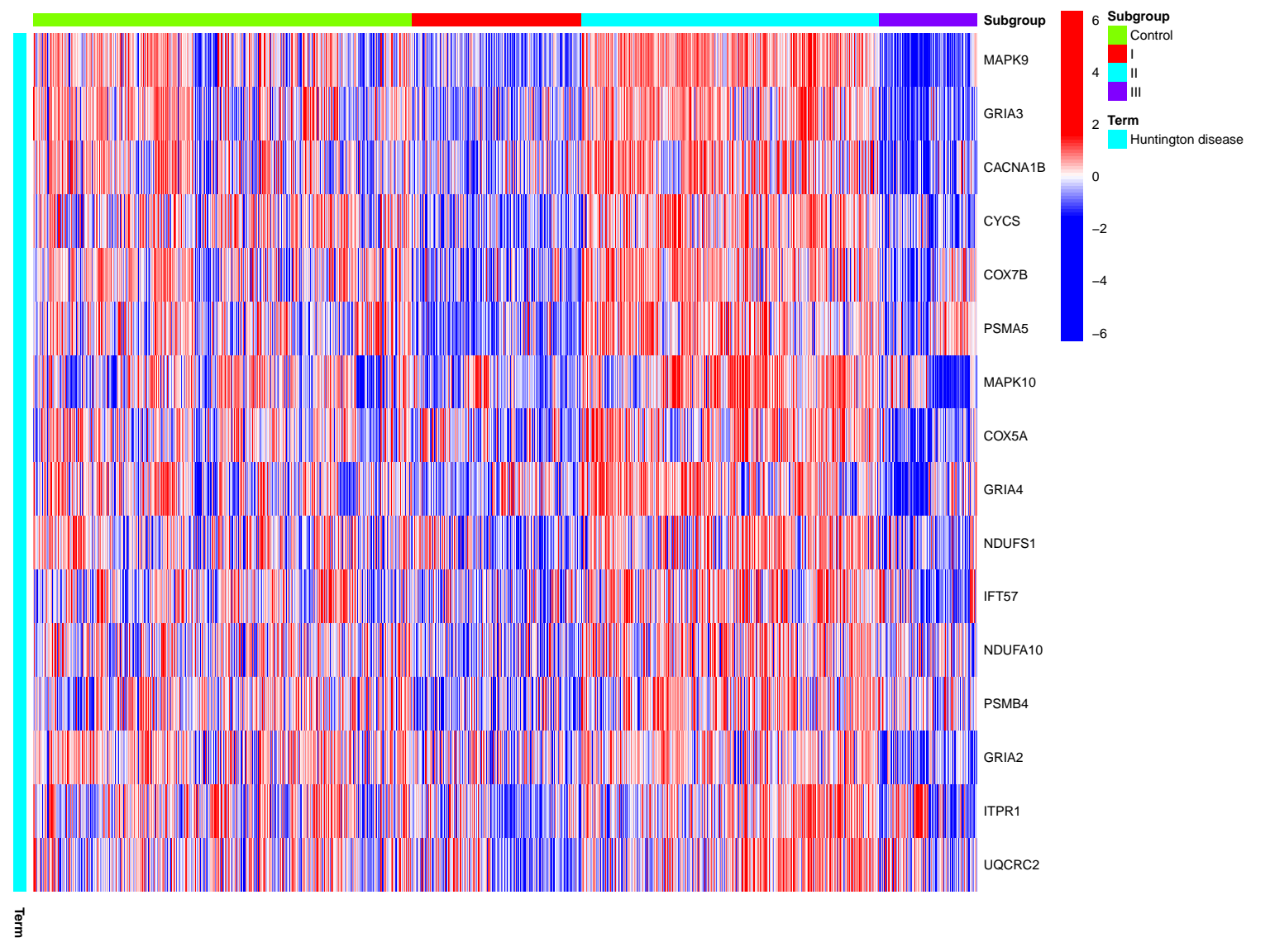

Supplement: Supplementary File 5 — Gene expression heatmap in each pathway. [file Data_Sheet_1.ZIP › Supplementary File 5.gene expression heatmap in each pathway/II-Huntington disease.pdf]

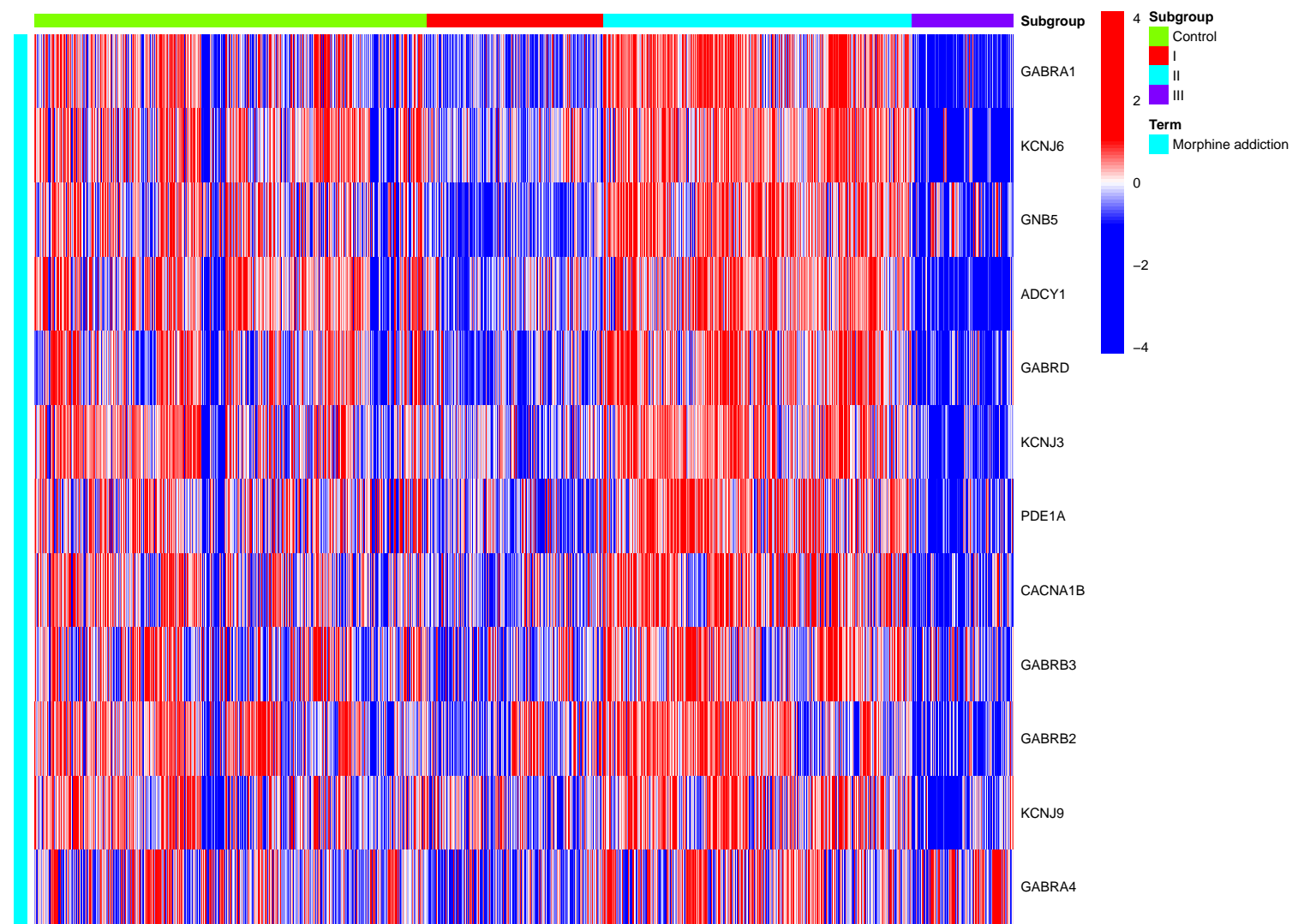

Term

Supplement: Supplementary File 5 — Gene expression heatmap in each pathway. [file Data_Sheet_1.ZIP › Supplementary File 5.gene expression heatmap in each pathway/II-Morphine addiction.pdf]

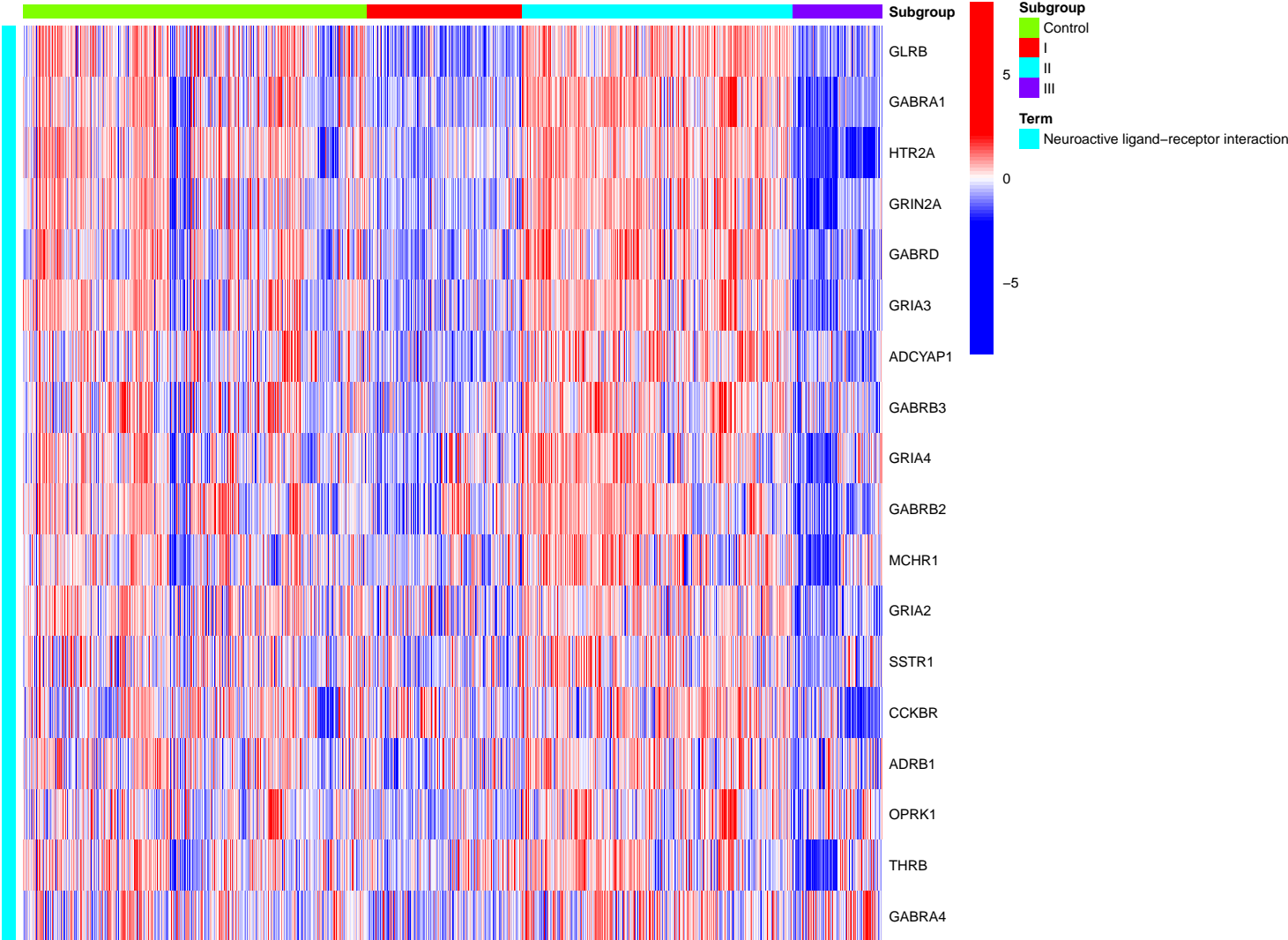

Term

Supplement: Supplementary File 5 — Gene expression heatmap in each pathway. [file Data_Sheet_1.ZIP › Supplementary File 5.gene expression heatmap in each pathway/II-Neuroactive ligand-receptor interaction.pdf]

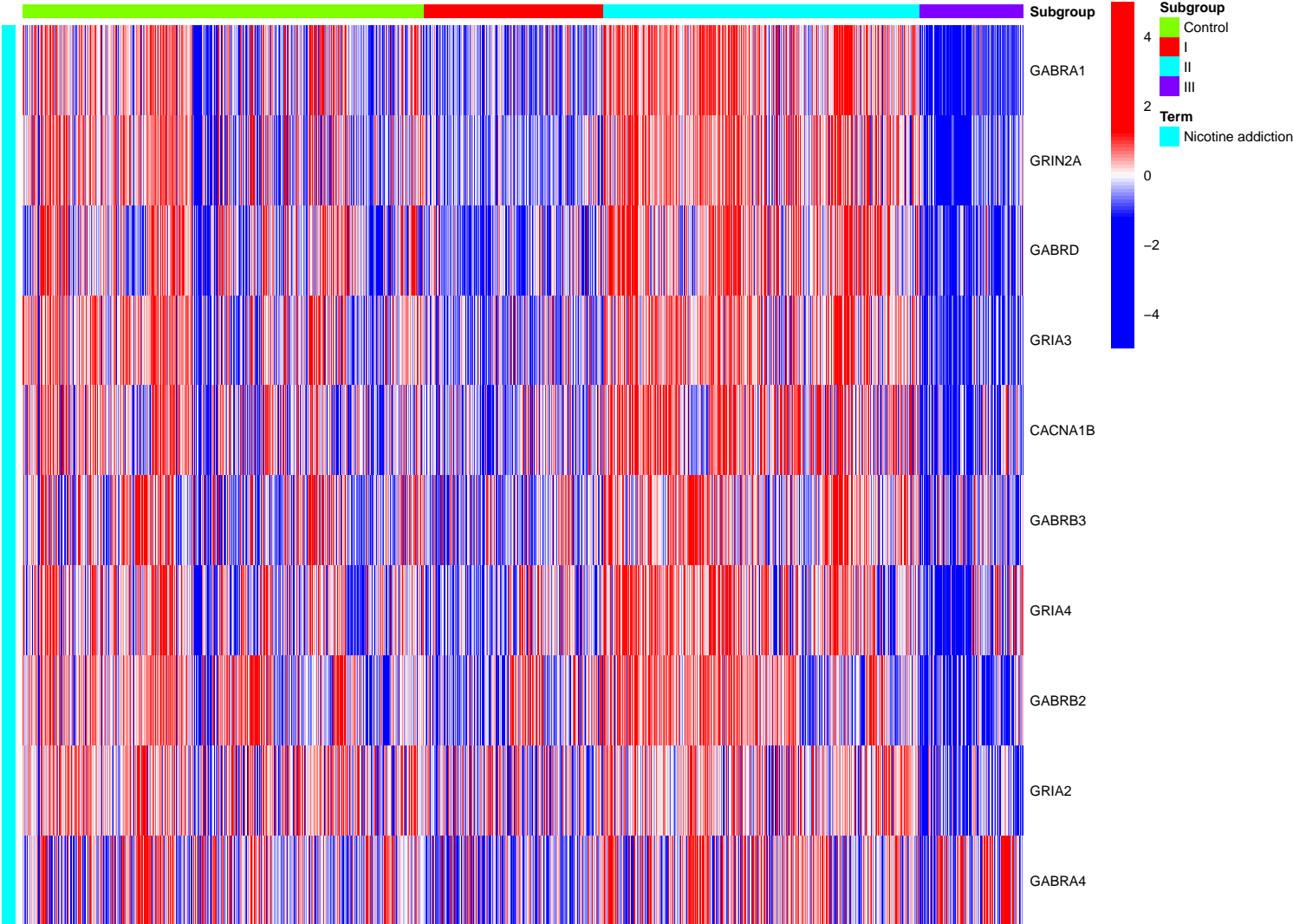

Term

Supplement: Supplementary File 5 — Gene expression heatmap in each pathway. [file Data_Sheet_1.ZIP › Supplementary File 5.gene expression heatmap in each pathway/II-Nicotine addiction.pdf]

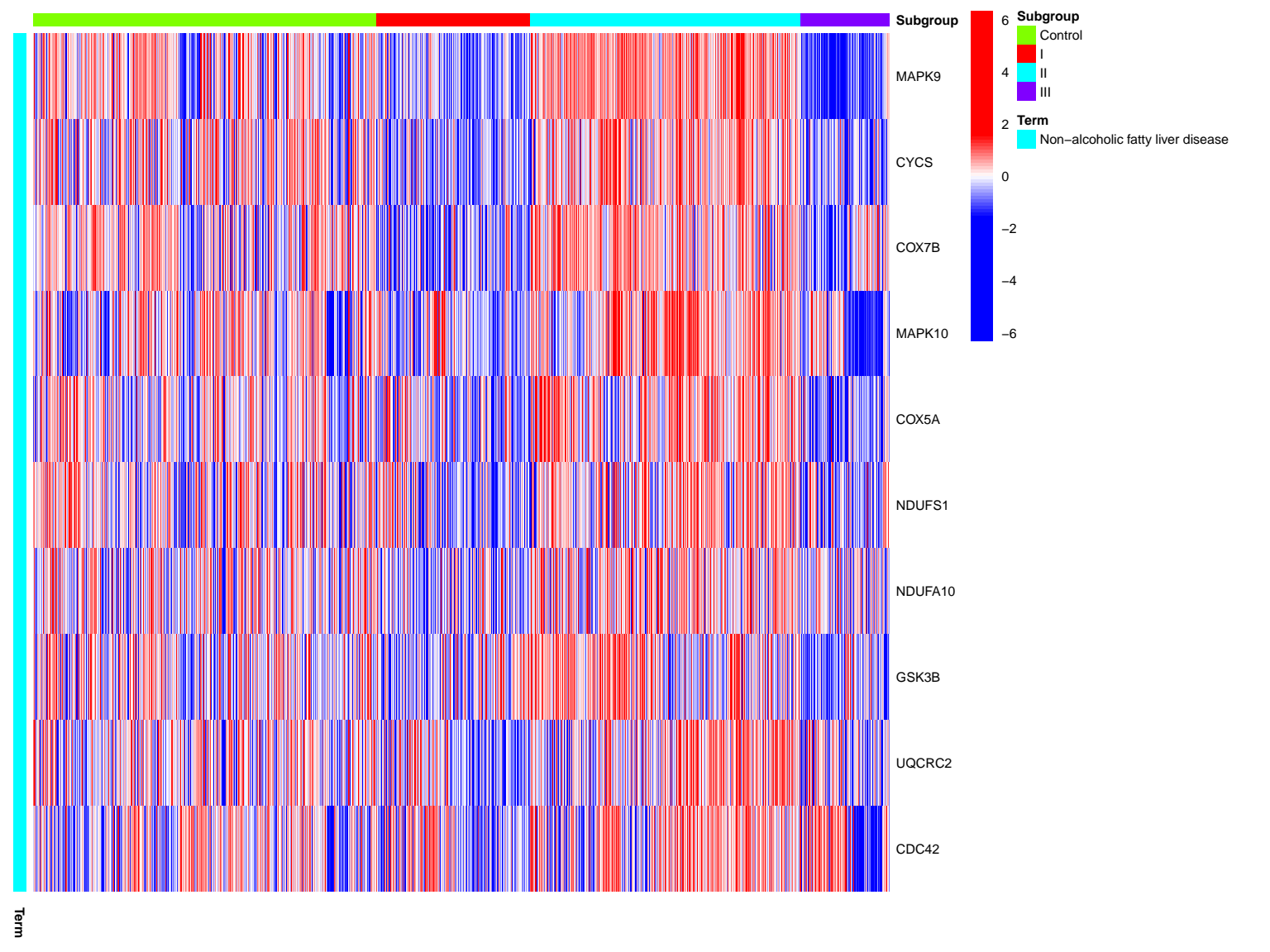

Supplement: Supplementary File 5 — Gene expression heatmap in each pathway. [file Data_Sheet_1.ZIP › Supplementary File 5.gene expression heatmap in each pathway/II-Non-alcoholic fatty liver disease.pdf]

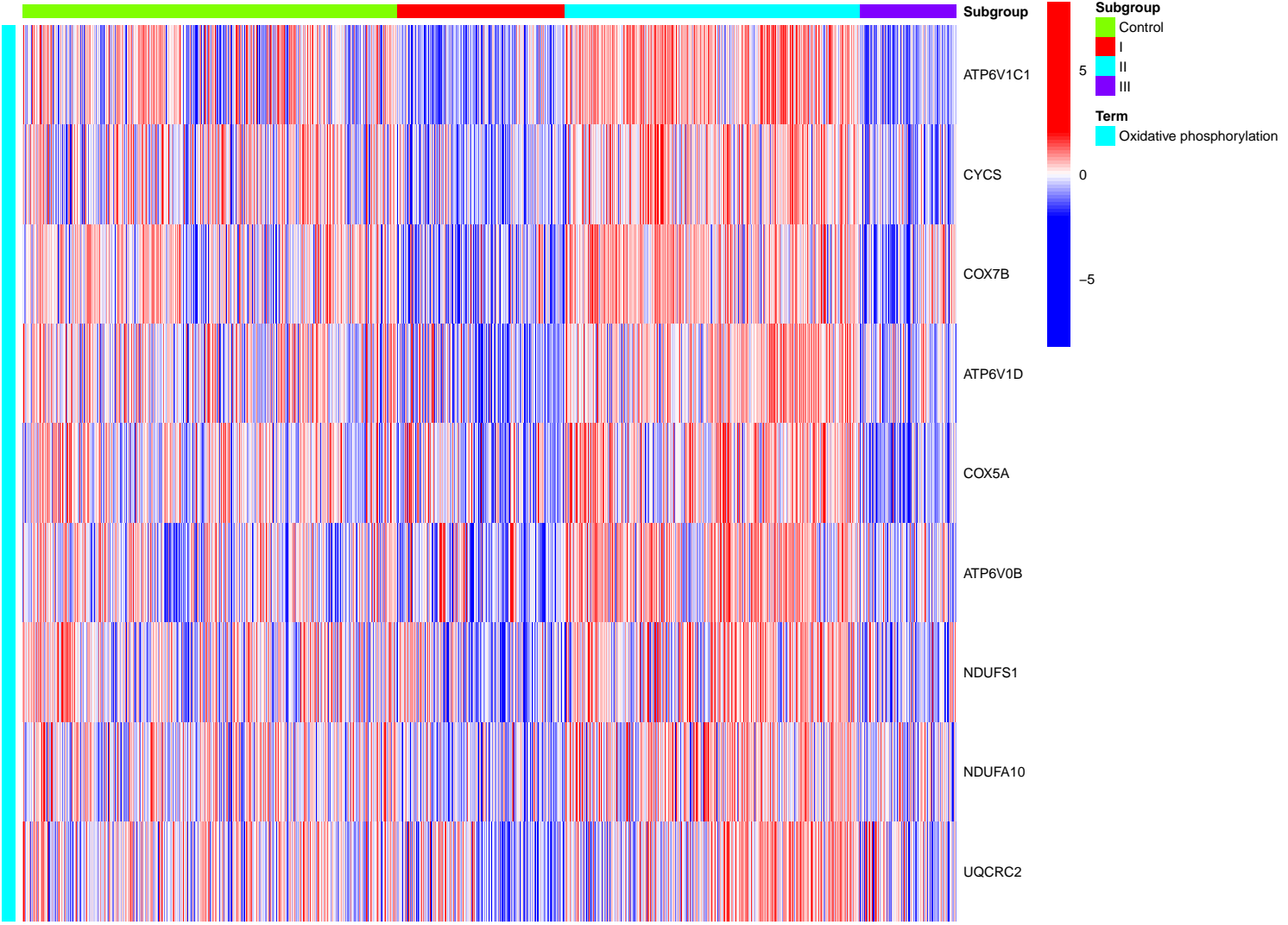

Term

Supplement: Supplementary File 5 — Gene expression heatmap in each pathway. [file Data_Sheet_1.ZIP › Supplementary File 5.gene expression heatmap in each pathway/II-Oxidative phosphorylation.pdf]

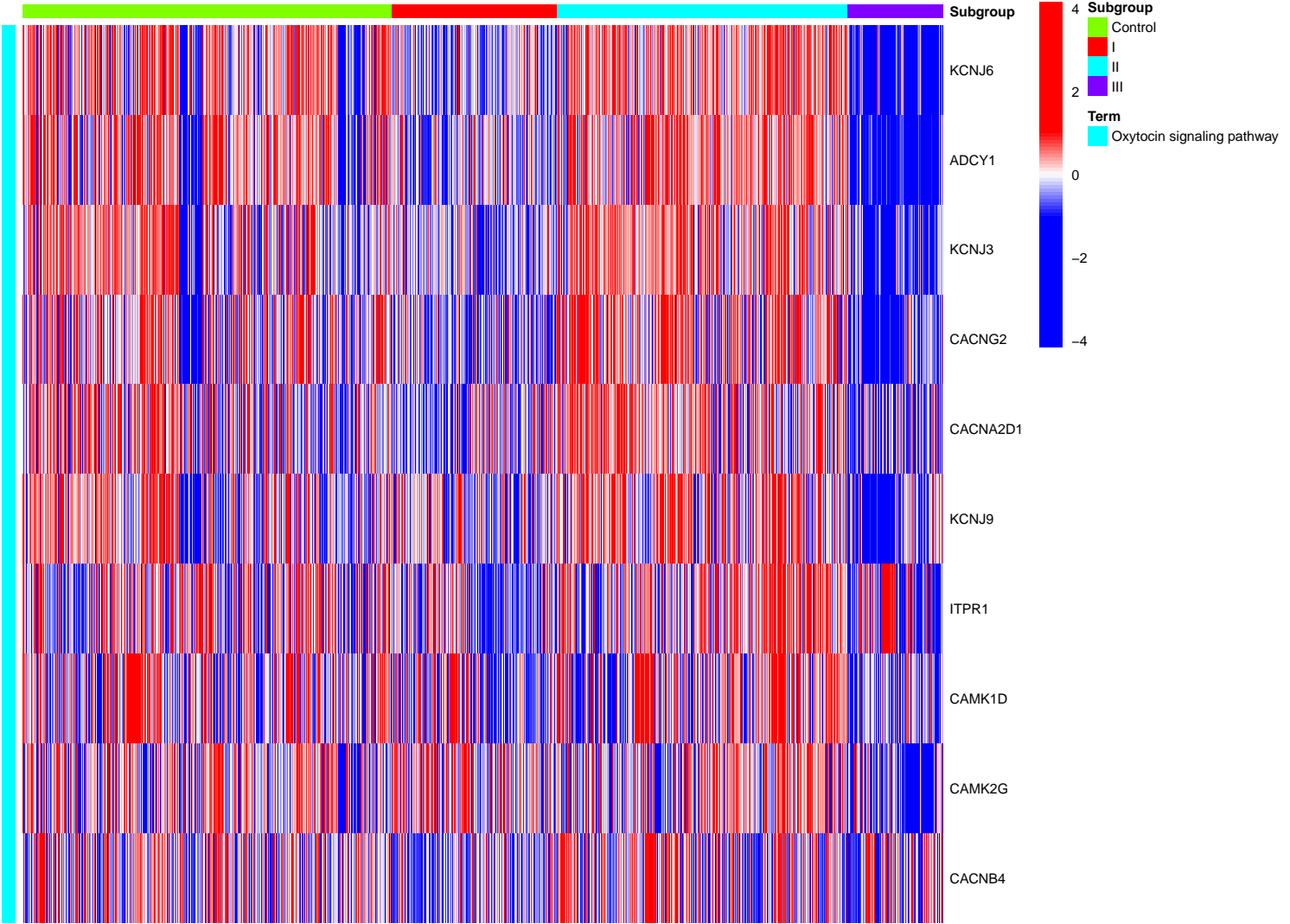

Term

Supplement: Supplementary File 5 — Gene expression heatmap in each pathway. [file Data_Sheet_1.ZIP › Supplementary File 5.gene expression heatmap in each pathway/II-Oxytocin signaling pathway.pdf]

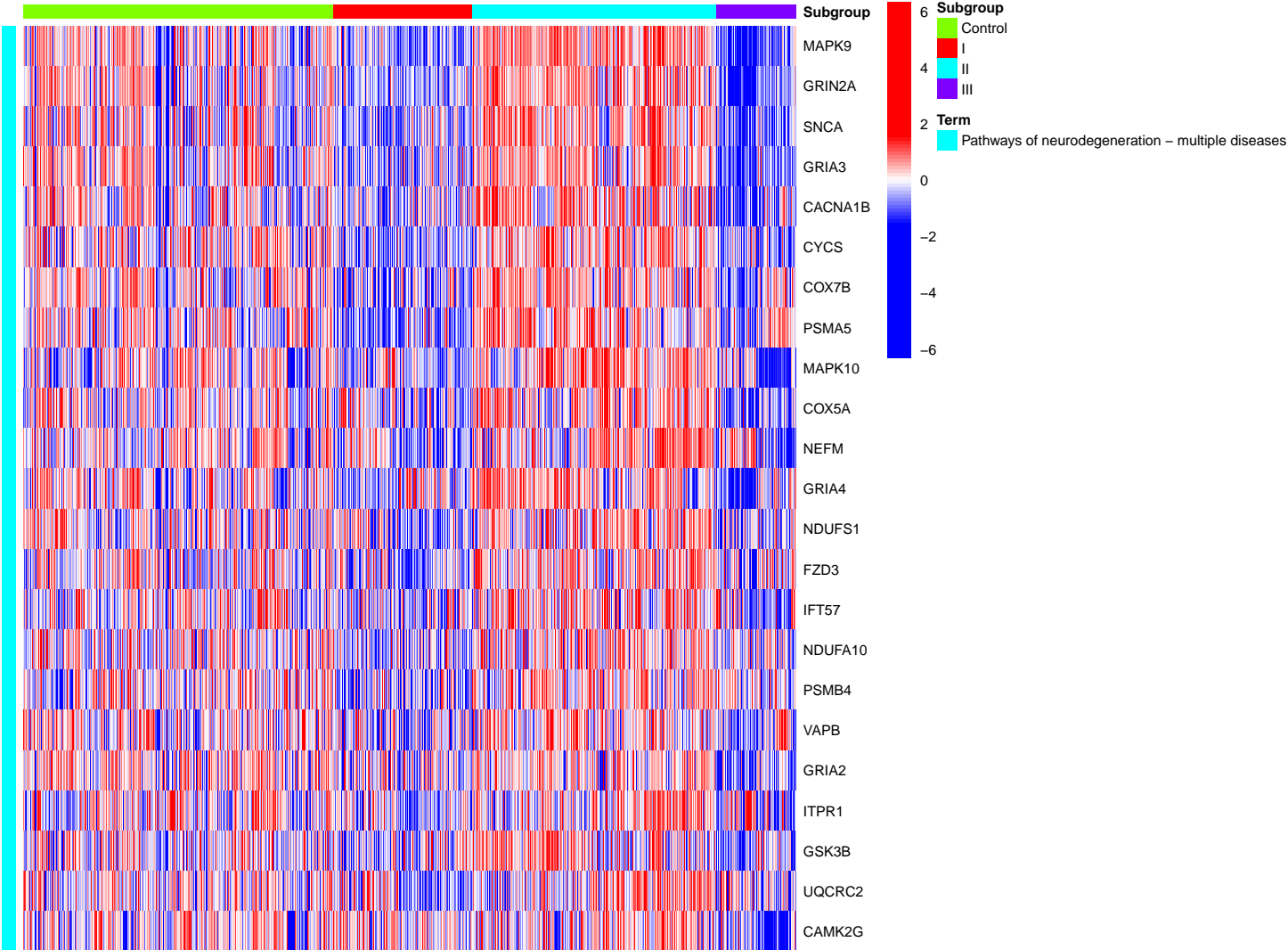

Term

Supplement: Supplementary File 5 — Gene expression heatmap in each pathway. [file Data_Sheet_1.ZIP › Supplementary File 5.gene expression heatmap in each pathway/II-Pathways of neurodegeneration - multiple diseases.pdf]

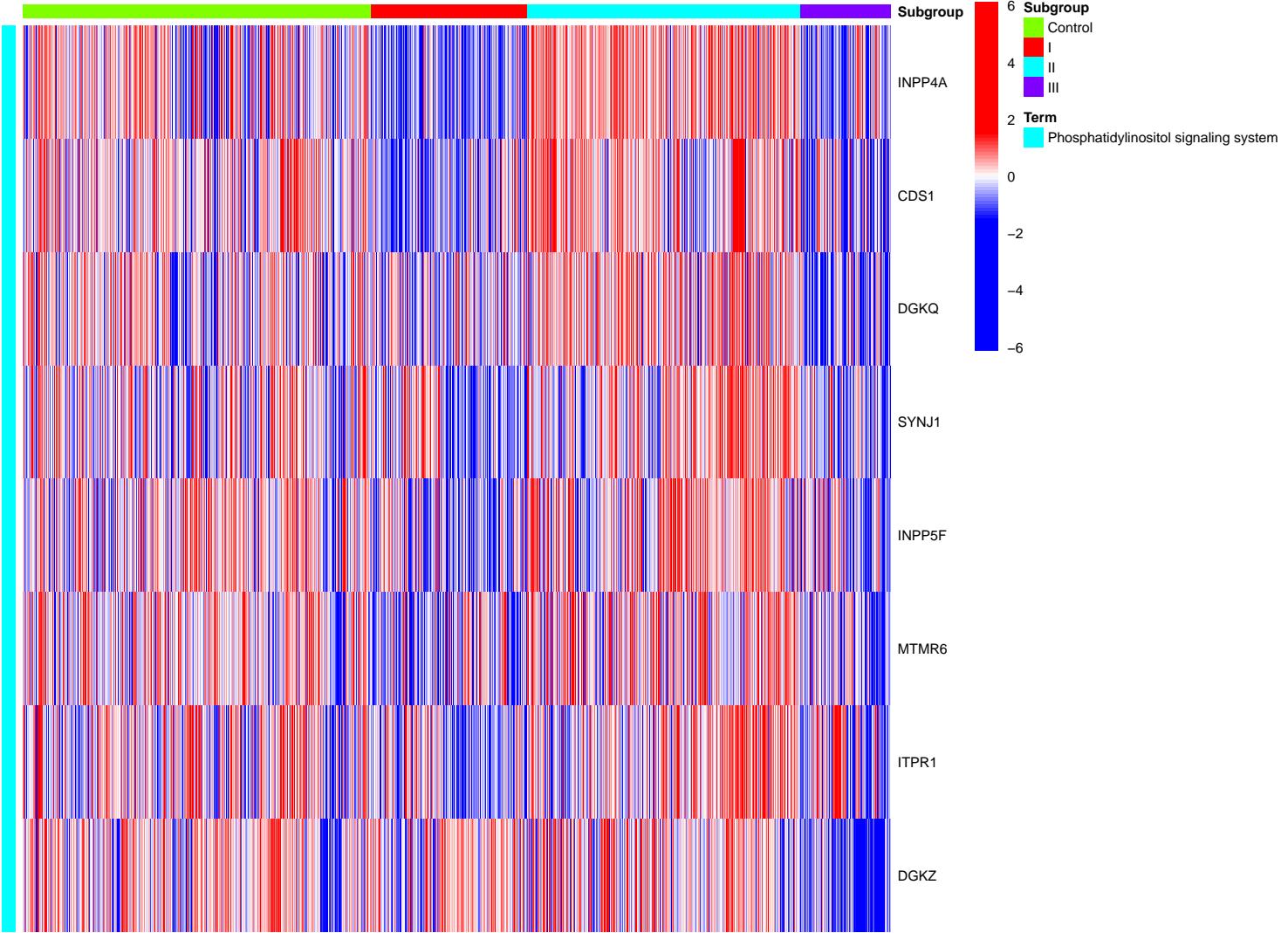

Term

Supplement: Supplementary File 5 — Gene expression heatmap in each pathway. [file Data_Sheet_1.ZIP › Supplementary File 5.gene expression heatmap in each pathway/II-Phosphatidylinositol signaling system.pdf]

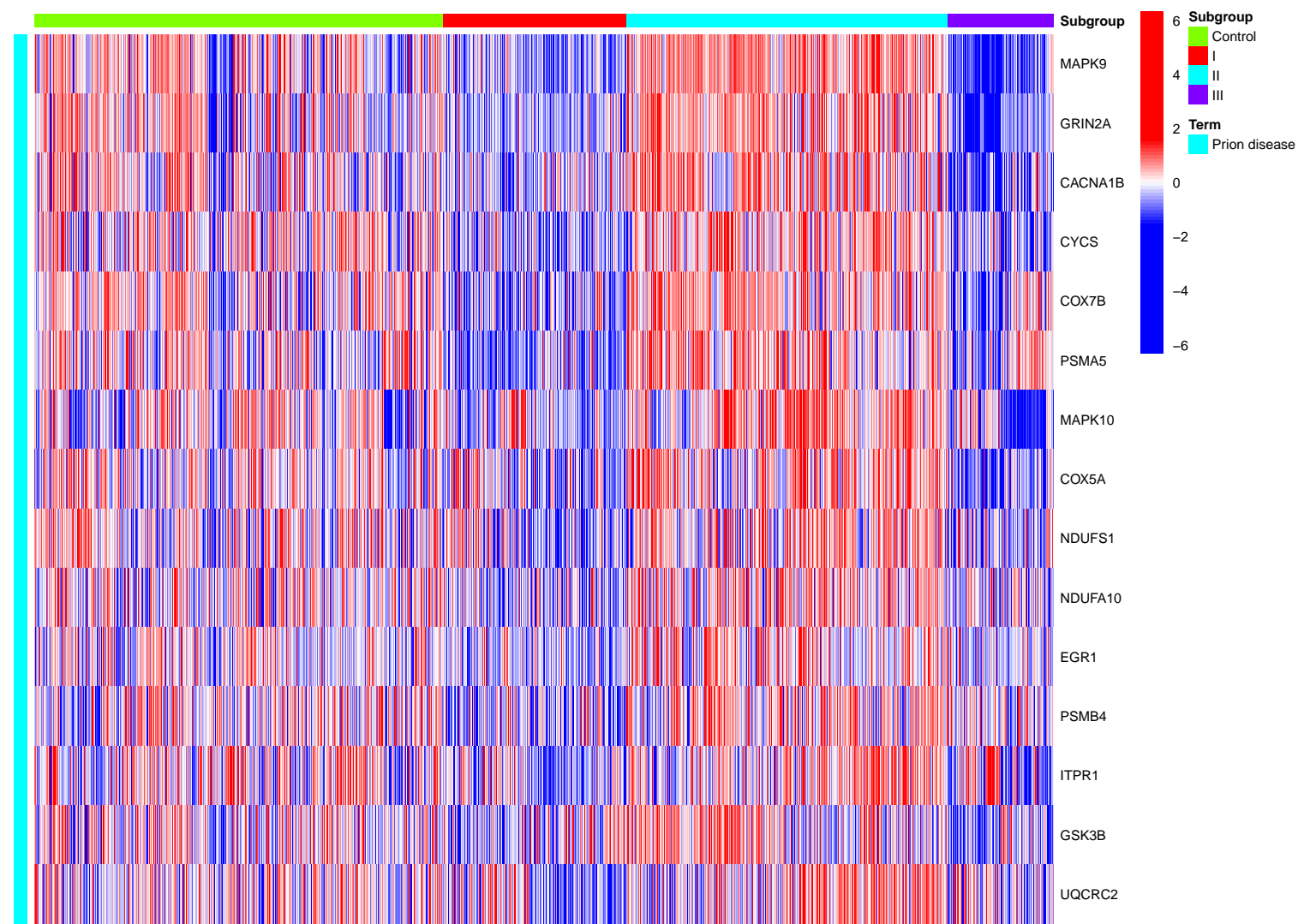

Term

Supplement: Supplementary File 5 — Gene expression heatmap in each pathway. [file Data_Sheet_1.ZIP › Supplementary File 5.gene expression heatmap in each pathway/II-Prion disease.pdf]

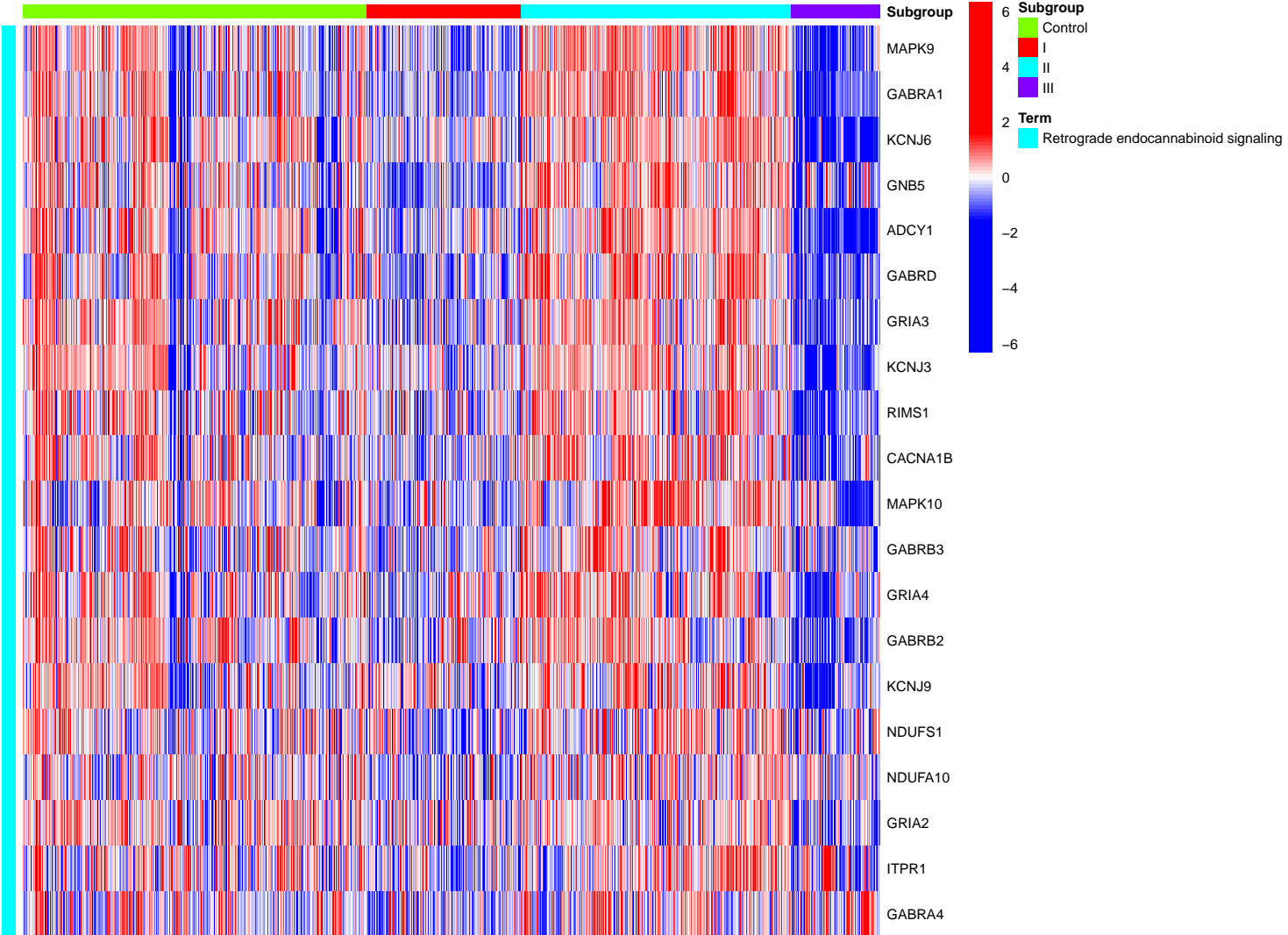

Term

Supplement: Supplementary File 5 — Gene expression heatmap in each pathway. [file Data_Sheet_1.ZIP › Supplementary File 5.gene expression heatmap in each pathway/II-Retrograde endocannabinoid signaling.pdf]

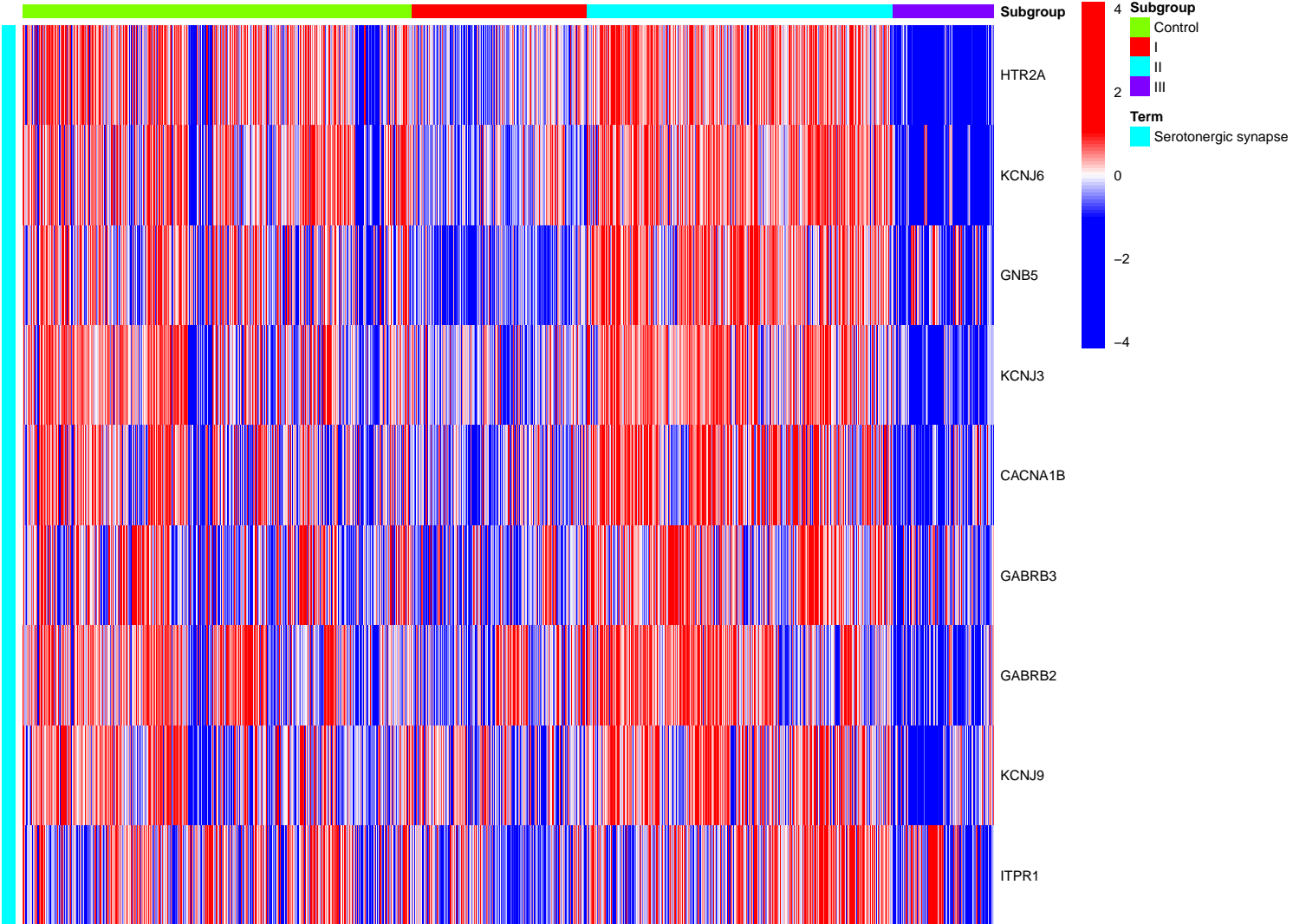

Term

Supplement: Supplementary File 5 — Gene expression heatmap in each pathway. [file Data_Sheet_1.ZIP › Supplementary File 5.gene expression heatmap in each pathway/II-Serotonergic synapse.pdf]

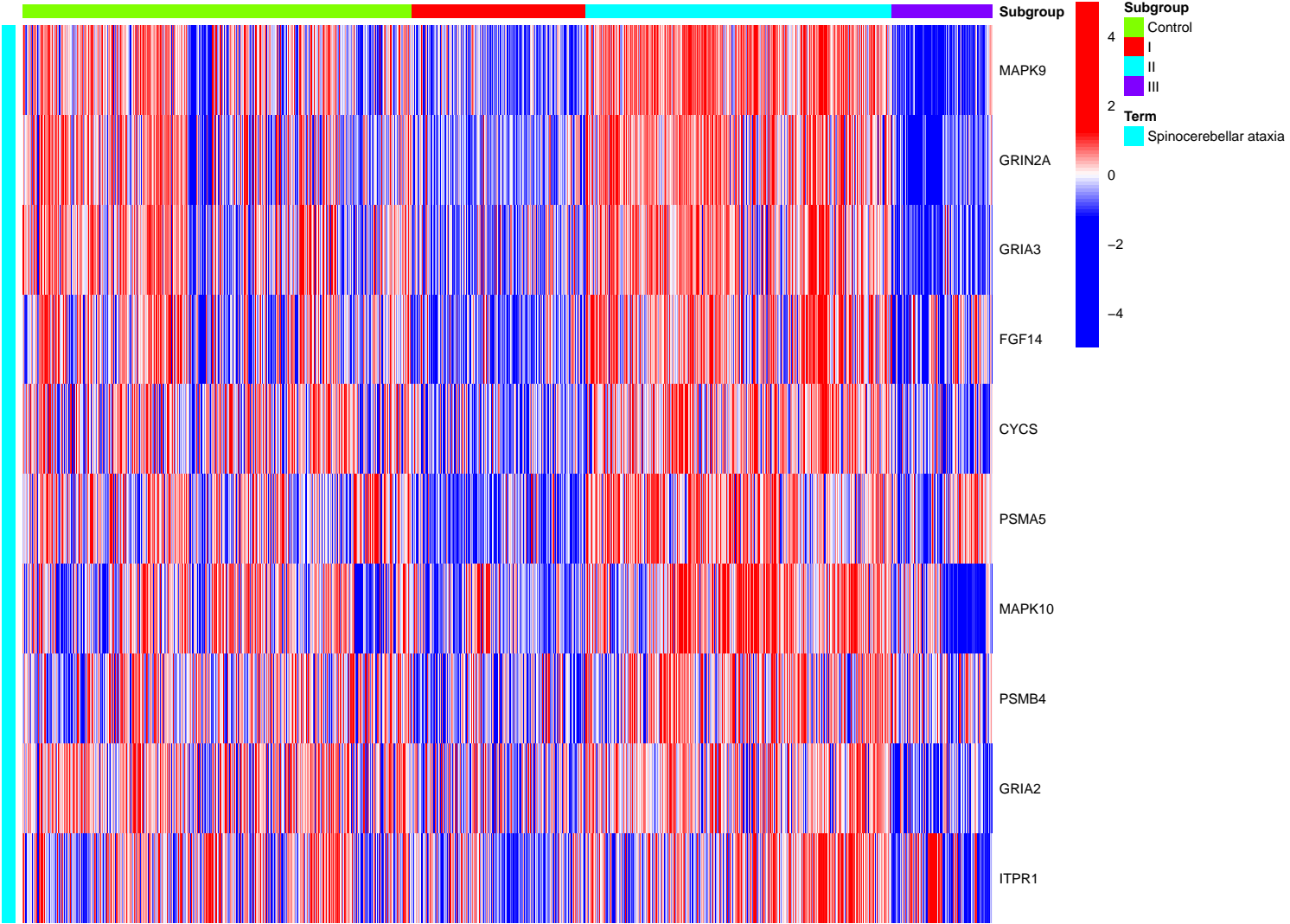

Term

Supplement: Supplementary File 5 — Gene expression heatmap in each pathway. [file Data_Sheet_1.ZIP › Supplementary File 5.gene expression heatmap in each pathway/II-Spinocerebellar ataxia.pdf]

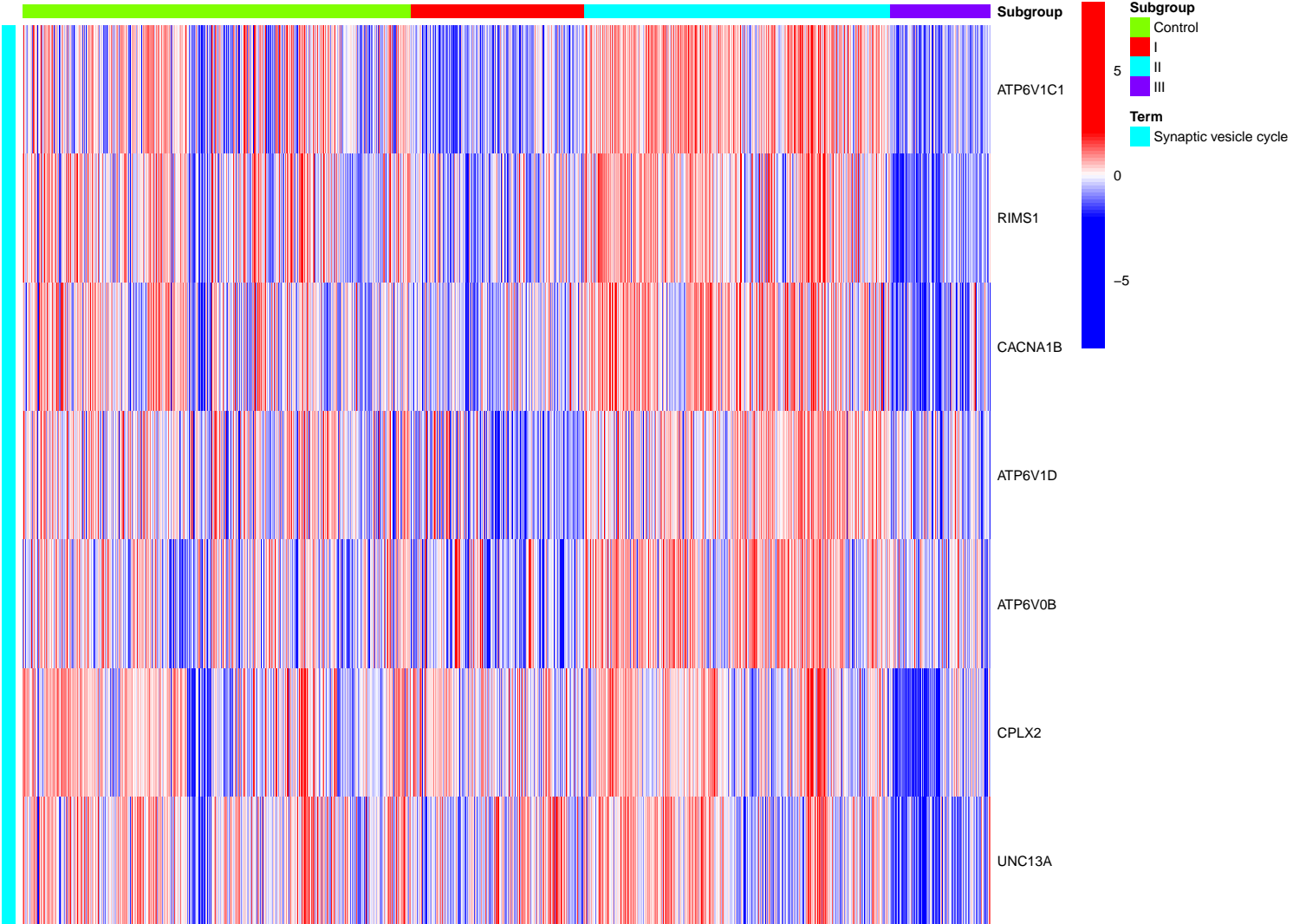

Term

Supplement: Supplementary File 5 — Gene expression heatmap in each pathway. [file Data_Sheet_1.ZIP › Supplementary File 5.gene expression heatmap in each pathway/II-Synaptic vesicle cycle.pdf]

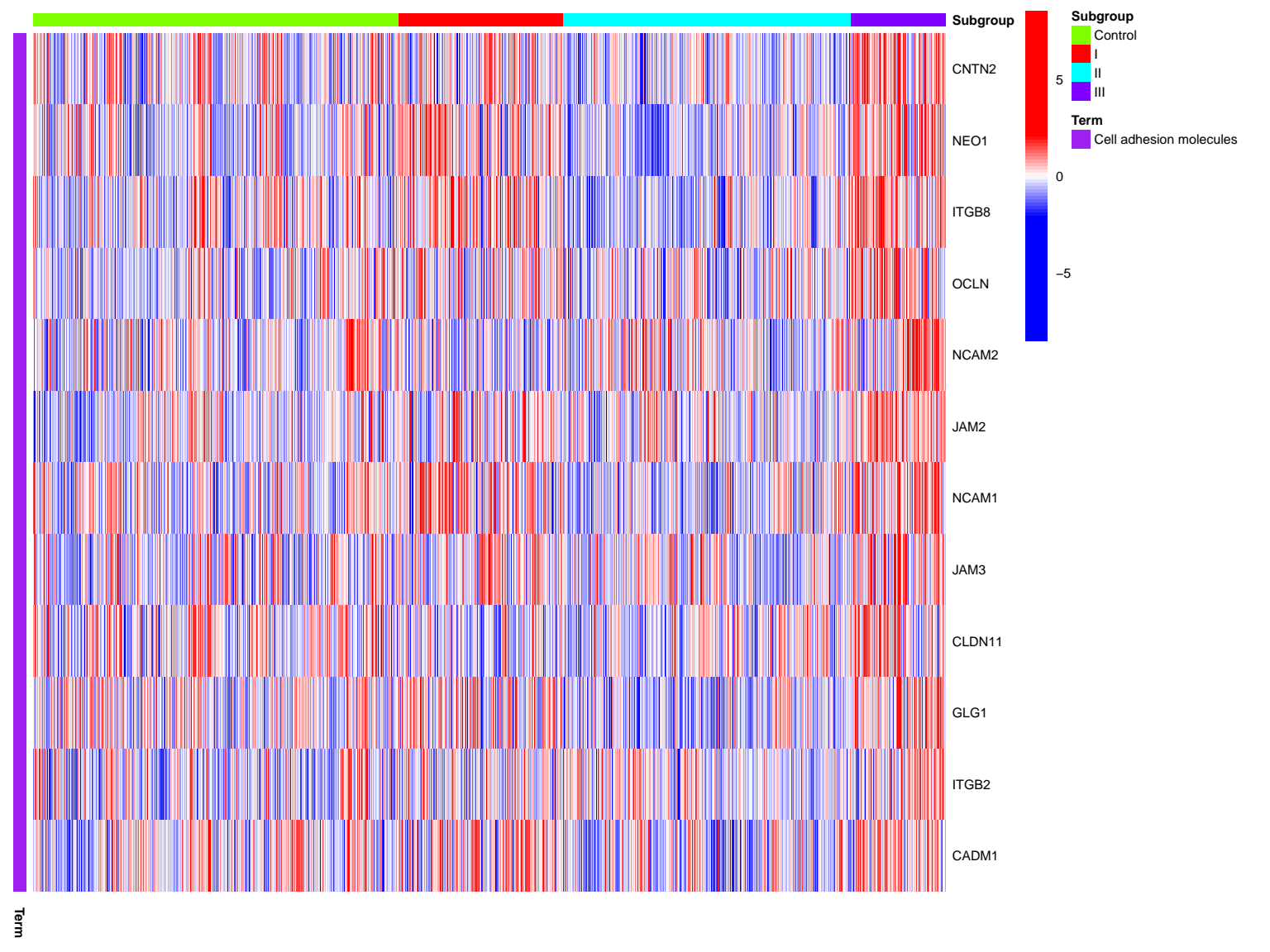

Supplement: Supplementary File 5 — Gene expression heatmap in each pathway. [file Data_Sheet_1.ZIP › Supplementary File 5.gene expression heatmap in each pathway/III-Cell adhesion molecules.pdf]

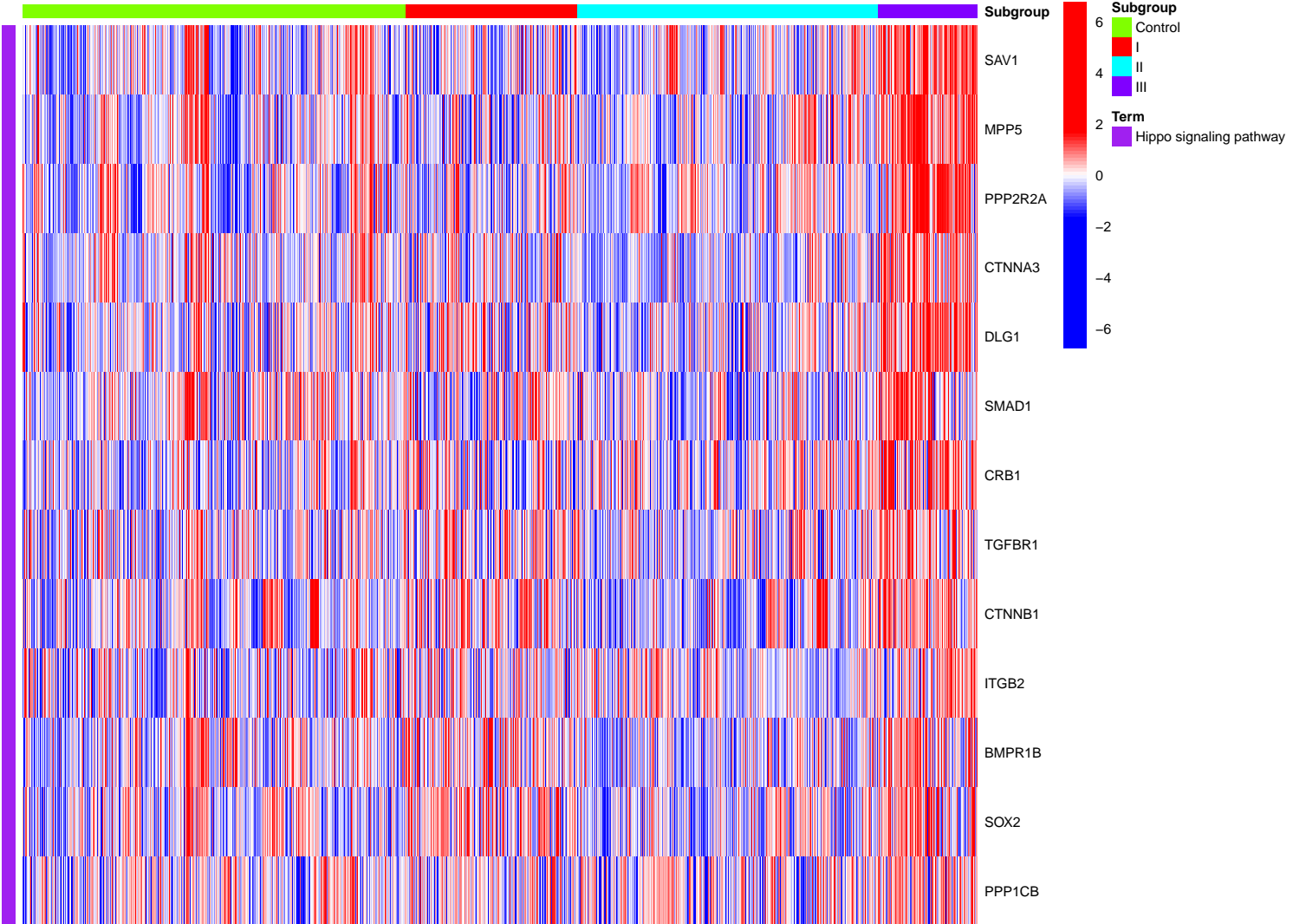

Term

Supplement: Supplementary File 5 — Gene expression heatmap in each pathway. [file Data_Sheet_1.ZIP › Supplementary File 5.gene expression heatmap in each pathway/III-Hippo signaling pathway.pdf]

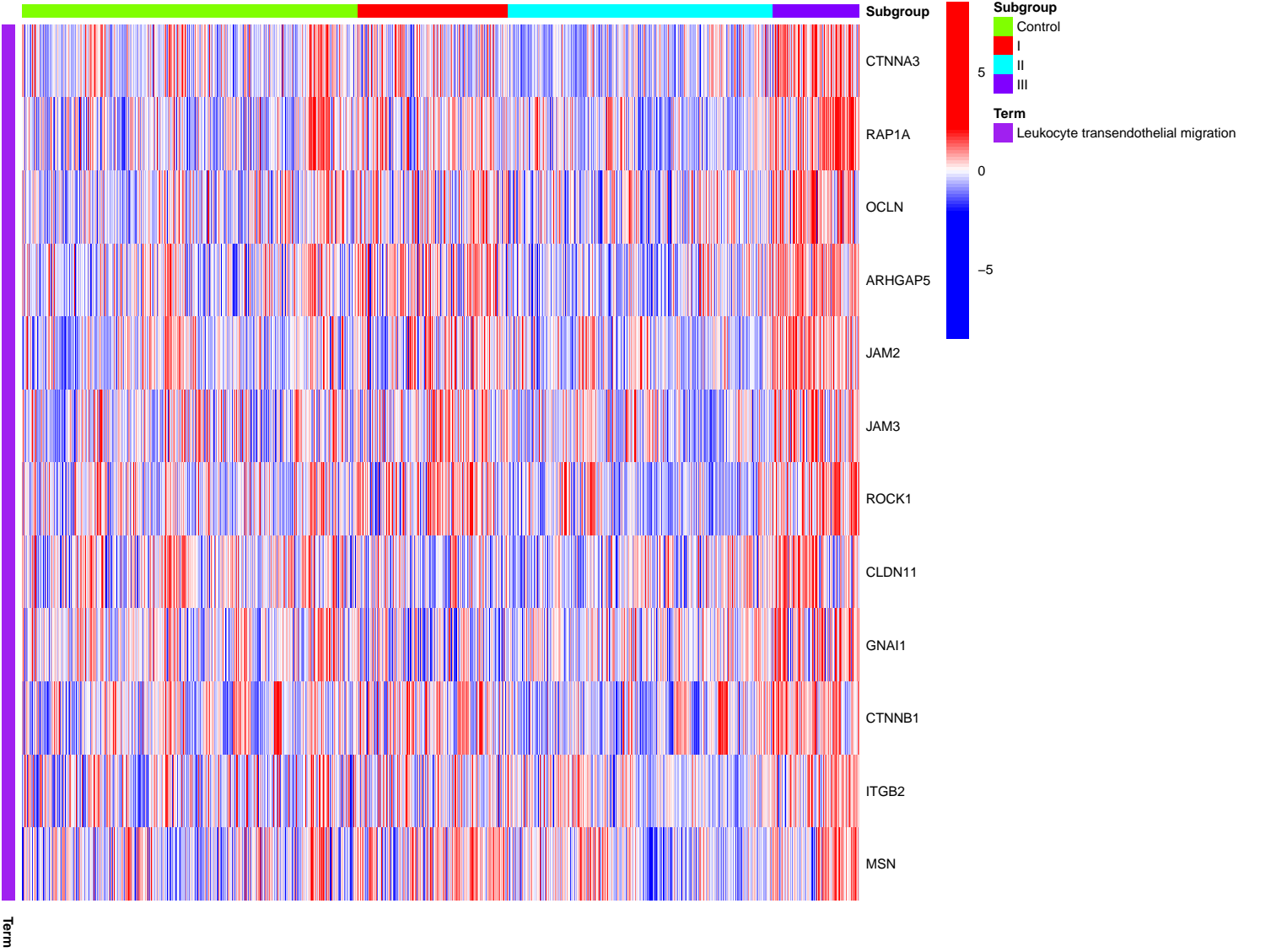

Supplement: Supplementary File 5 — Gene expression heatmap in each pathway. [file Data_Sheet_1.ZIP › Supplementary File 5.gene expression heatmap in each pathway/III-Leukocyte transendothelial migration.pdf]

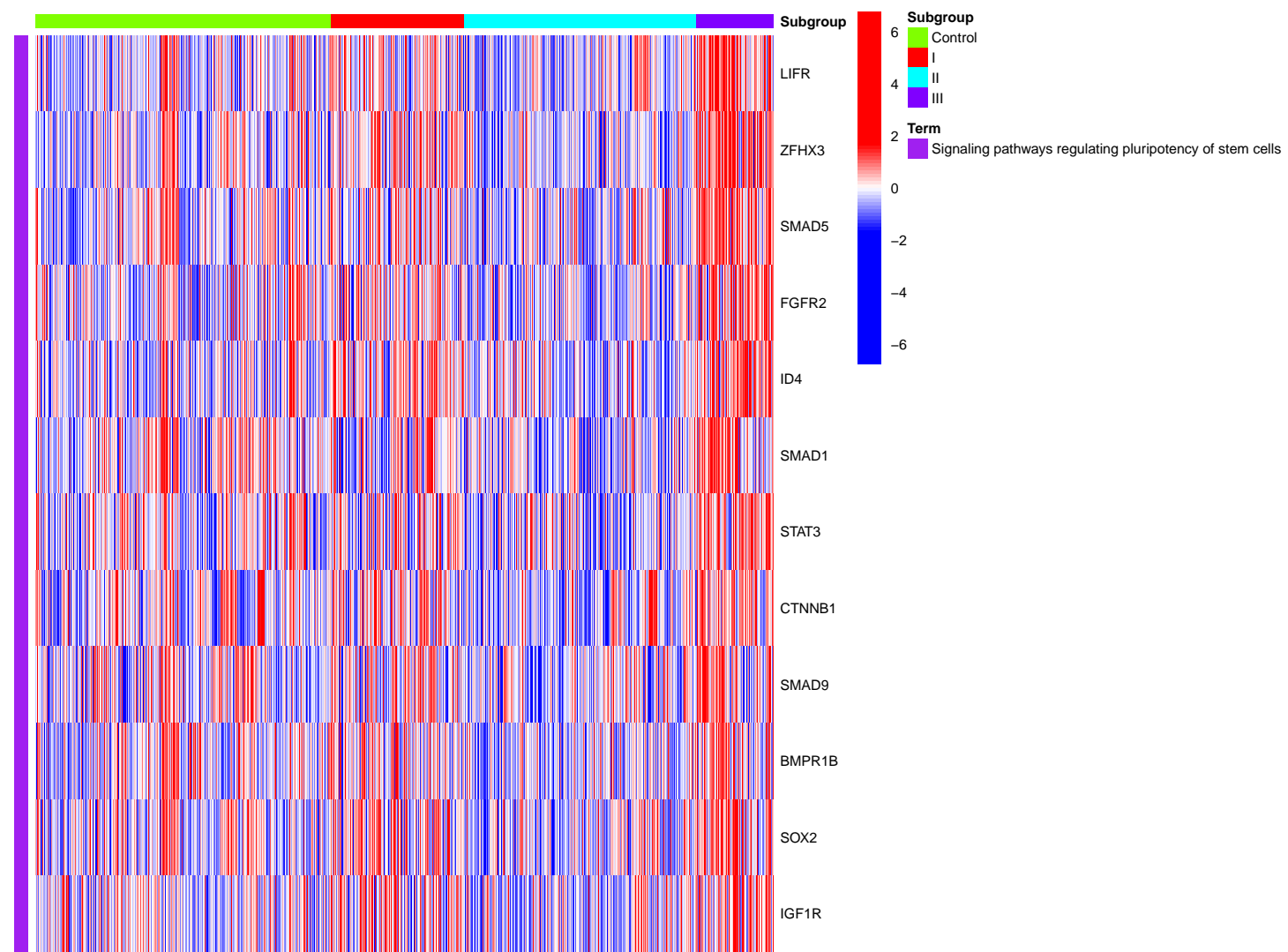

Term

Supplement: Supplementary File 5 — Gene expression heatmap in each pathway. [file Data_Sheet_1.ZIP › Supplementary File 5.gene expression heatmap in each pathway/III-Signaling pathways regulating pluripotency of stem cells.pdf]

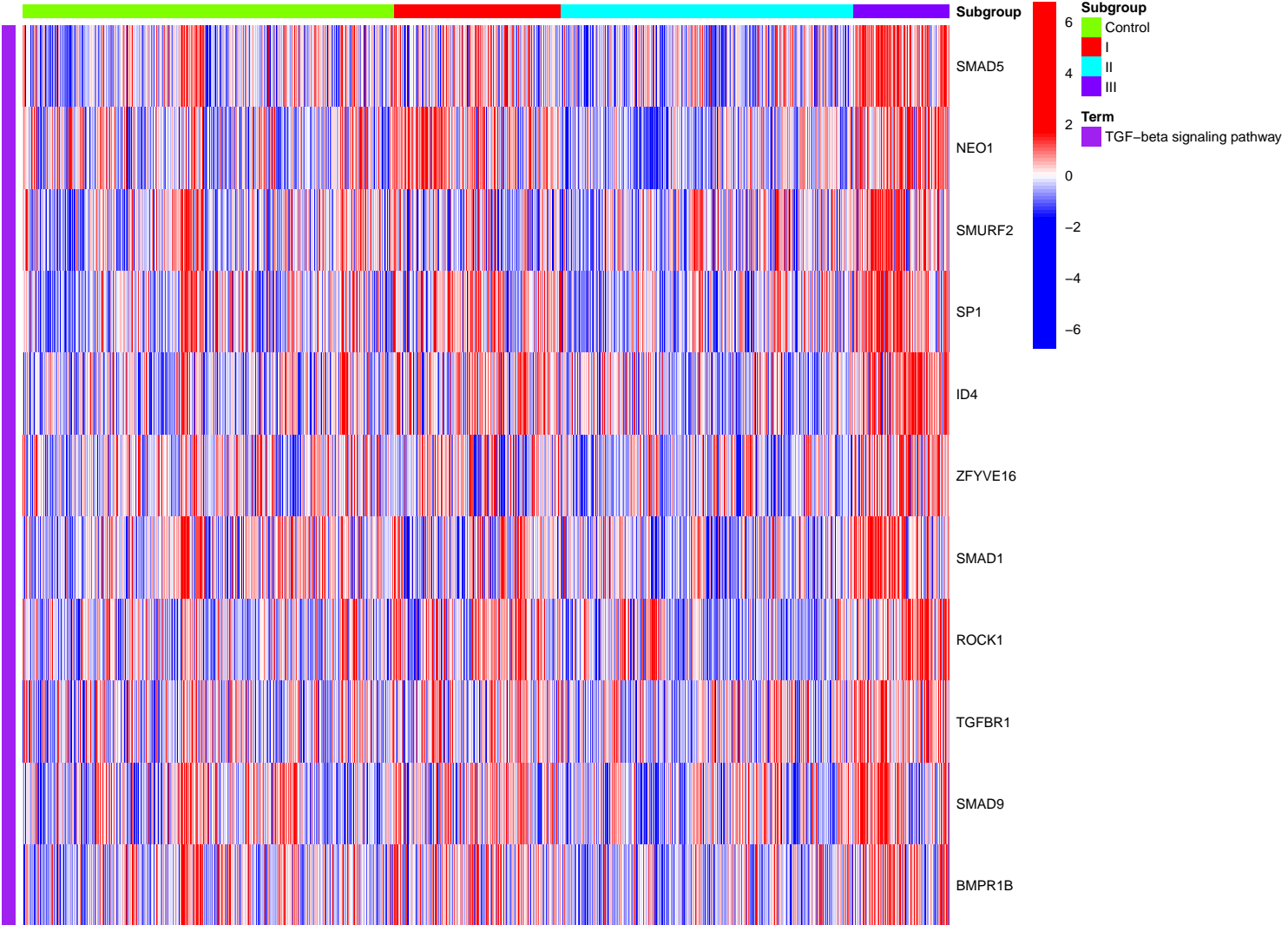

Supplement: Supplementary File 5 — Gene expression heatmap in each pathway. [file Data_Sheet_1.ZIP › Supplementary File 5.gene expression heatmap in each pathway/III-TGF-beta signaling pathway.pdf]

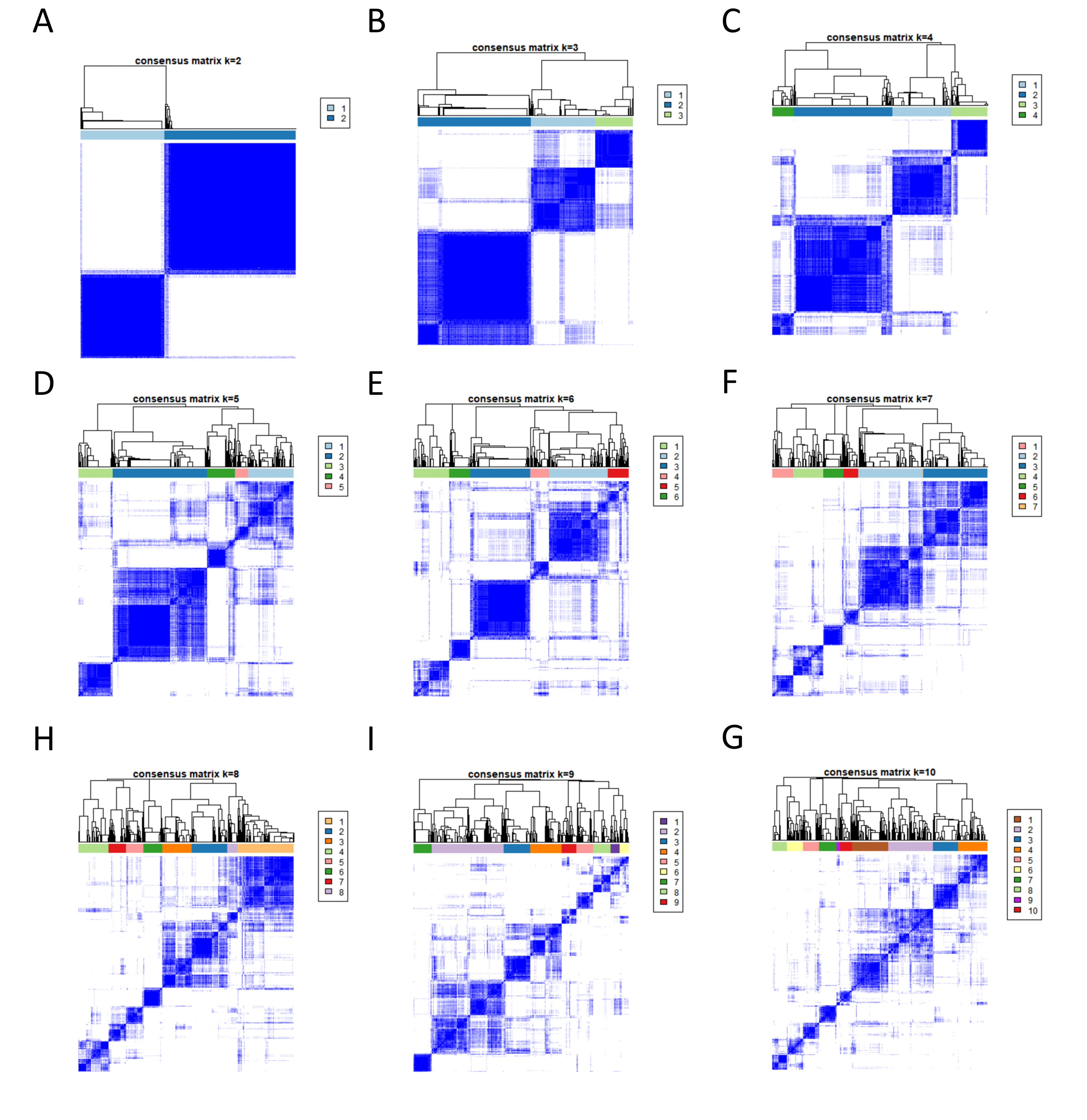

Supplement: Supplementary file 9 [file Image_1.TIF]
