# Supplementary material for: Identification of the molecular subgroups in Alzheimer's disease by transcriptomic data
Source: Front Neurol. 2022 Sep 20;13:901179. doi: 10.3389/fneur.2022.901179 (PMC9530954; doi:10.3389/fneur.2022.901179)
Supplement: Supplementary File 8 — List of major acronyms. [file Table_7.DOCX]

| Full title | Abbreviation |
| --- | --- |
| Alzheimer's disease | AD |
| central nervous system | CNS |
| Clinical Dementia Rating Scale | CDR |
| Neurofibrillary tangles | NFT |
| Neuritic Plaque Density | NPD |
| gene set enrichment analysis | GSEA |
| presenilin 1 | PSEN1 |
| long-term potentiation | LTP |
| N-methyl-d-aspartate receptor | NMDAR |
| γ -aminobutyric acid receptor A | GABAAR |
| brain-derived neurotrophic factors | BDNF |
| amyloid precursor protein | APP |
| Receiver operating characteristic | ROC |
| areas under the curve | AUC |

List of major acronyms
